# Glycerol Electro‐Oxidation to Dihydroxyacetone with Coupled Hydrogen Production via In Situ Optimization of Water Oxidation Intermediates

**DOI:** 10.1002/smll.202504892

**Published:** 2025-08-04

**Authors:** Sandip K. Pahari, Heng Jao, Chih‐Chian Chang, Kuan‐Wei Su, Yung‐Fu Chen, Yit‐Tsong Chen

**Affiliations:** ^1^ Department of Electrophysics PSMC‐NYCU Research Center, and LIGHTMED Laser System Research Center National Yang Ming Chiao Tung University Hsinchu 300093 Taiwan; ^2^ Department of Chemistry National Taiwan University Taipei 10617 Taiwan

**Keywords:** 1, 3‐dihydroxyacetone, bismuth‐doped cobalt oxide, glycerol oxidation reaction, in situ Raman spectroscopy, water oxidation intermediates

## Abstract

In electrocatalytic water splitting, the selective oxidation of glycerol (GLY) to 1,3‐dihydroxyacetone (DHA) presents a promising alternative to the oxygen evolution reaction (OER) and enables the concurrent production of valuable chemicals and hydrogen. However, controlling this selective oxidation is challenging due to similar reactivities of the hydroxyl groups of GLY. In this study, an electrocatalyst is synthesized by combining phosphated few‐layer phosphorene (FLP‐P) with bismuth‐doped cobalt oxide (Bi‐Co_3_O_4_). In the anodic reactions, the Bi center coordinates selectively with the secondary hydroxyl group of GLY; Co_3_O_4_ forms the OER intermediates, and the phosphate groups on FLP‐P stabilize the OER intermediates through a bifunctional mechanism. In situ Raman spectroscopy is employed to optimize the production of the OER intermediates for achieving 85% GLY conversion reaction and 89% DHA product selectivity in neutral medium. The simultaneous production of valuable chemicals and high‐purity hydrogen exemplifies the advancement of green hydrogen production in water electrolysis.

## Graphical abstract

A multifunctional electrocatalyst is developed for selective glycerol oxidation to dihydroxyacetone and hydrogen evolution simultaneously. Herein, a bifunctional mechanism aided water oxidation intermediates stabilization with in situ Raman spectroscopic determination of optimum formation condition of those intermediates and selective coordination of glycerol with electrocatalyst, enables dihydroxyacetone conversion and selectivity.

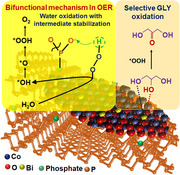

## 1 Introduction

Glycerol (GLY) is an abundantly available biomass platform molecule that can be oxidized to a number of industrially important aldehydes, ketones, and carboxylates.^[^ ^1^ ^]^ Among the oxidation products of GLY, 1,3‐dihydroxyacetone (DHA) is regarded as one of the most valuable compounds due to its wide use in pharmaceutics, cosmetics, biopolymers, and fine chemical industry.^[^ ^2^, ^3^ ^]^ However, the formation of DHA through the selective oxidation of the secondary hydroxyl group (−OH) of GLY is highly challenging, because the thermodynamic barriers of oxidizing the (middle) secondary and the other two (terminal) primary hydroxyl groups of GLY are very close. As a result, the oxidation of GLY usually involves inevitable competition between the terminal and middle C─OH or C─C oxidative cleavages, multiple reaction paths, and various oxidation products (see the details outlined in Figure S1, Supporting Information).^[^ ^4^ ^]^

For the selective GLY oxidation to DHA, the methods like microbial fermentation and chemical oxidation were mainly employed, but suffered from high production cost, long operation times, and harsh reaction conditions.^[^ ^5^ ^]^ In this context, the electrochemical GLY oxidation approach, which operates efficiently under the mild and environmentally benign conditions without reliance on hazardous chemical oxidants, has emerged as a compelling alternative to traditional oxidative strategies. Moreover, the process can be synergistically coupled with advantageous cathodic reactions − such as hydrogen evolution or CO_2_ reduction − thereby enhancing the general economic viability and sustainability of the overall process.^[^ ^6^, ^7^, ^8^ ^]^ Most studies on the GLY electro‐oxidation have so far centered on using precious metals as catalysts, such as gold (Au), platinum (Pt), palladium (Pd), and their metal alloys.^[^ ^9^ ^]^ It has been demonstrated that the noble metal catalysts doped with bismuth (Bi), or antimony (Sb), can promote the selective adsorption of the secondary hydroxyl group of GLY on the Bi or Sb center, leading to a highly selective oxidation of GLY to DHA.^[^ ^10^, ^11^ ^]^ Despite noble metal catalysts for high DHA selectivity of the GLY electro‐oxidation, their high cost, slow reaction kinetics, CO poisoning, active site blockage, and low current densities have resulted in reducing DHA yield and limiting practical applications.^[^ ^11^ ^]^ To address these challenges, non‐noble metal electrocatalysts, particularly the Ni‐based ones, have been widely used, where NiOOH, serving as the active catalyst layer, can oxidize alcohols through two electrochemical dehydrogenation mechanisms.^[^ ^12^ ^]^ Alternatively, cupric oxide (CuO) was demonstrated to be able to oxidize the secondary hydroxyl group of GLY, giving rise to a high DHA yield at higher current densities (3 mA cm^2^), compared to Pt/C (10^−1^ mA cm^2^), under various pH conditions. It was also shown that the DHA production is favored at pH 9, whereas at pH 13, DHA converts spontaneously to glyceraldehyde (GLD) without the need for an applied potential.^[^ ^13^ ^]^ Moreover, manganese oxide (MnO_2_) shows high DHA selectivity in 0.1 m Na_2_B_4_O_7_ electrolyte solutions, of which the selectivity toward DHA can be improved as the applied potential increases.^[^ ^14^ ^]^ Amorphous cobalt oxide (CoO_x_) has been demonstrated to oxidize GLY efficiently with excellent DHA selectivity due to its greater surface defects and randomly oriented bonds when compared to crystalline oxides.^[^ ^15^ ^]^ In another work, Bi‐doped spinel Co_3_O_4_ with the hydroxyl species (OH* from water oxidation) as an oxidant was used to accelerate the oxidation of the hydroxyl groups of GLY and promote the carbon‐carbon bond cleavage, resulting in the high formate selectivity of GLY oxidation.^[^ ^16^ ^]^

From previous studies, it is known that the selective coordination of GLY with electrocatalysts, the nature of oxidants, applied potential, and the pH of electrolytes all play significant roles in the selective oxidation of GLY to DHA. For instance, at high pH, the hydroxide ions (OH^−^) can reduce the activation barrier for oxidizing GLY to an aldehyde/ketone intermediate through dehydrogenation. However, this intermediate can further undergo the β‐hydride elimination by OH^−^, leading to the formation of a carboxylic acid. As a result, despite the accelerated glycerol oxidation reaction (GOR) at high pH, the negative effect of overoxidation impacts the selective production of DHA. Therefore, the electro‐oxidation of GLY in neutral pH solutions without the involvement of hydroxyl radicals may be the key factor to improve DHA selectivity.^[^ ^17^ ^]^ Although the Bi‐based catalysts have been shown to enhance DHA selectivity in GOR, likely due to preferential coordination with the secondary hydroxyl group of GLY, the direct spectroscopic evidence for the role of Bi in the reaction mechanism is still sparse.^[^ ^10^ ^]^

**Figure 1:**
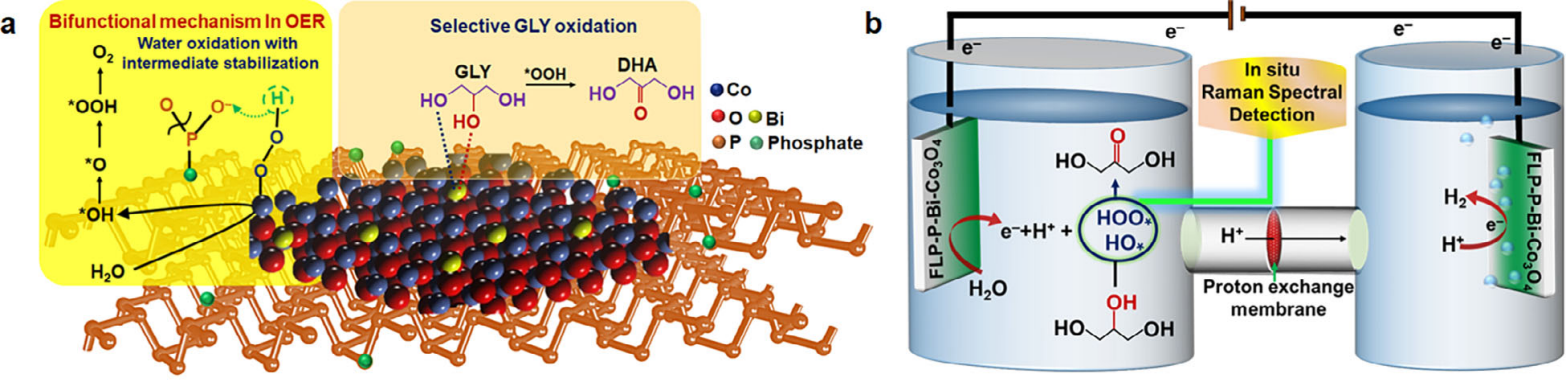
The design concept of an FLP‐P‐Bi‐Co_3_O_4_ hybrid electrocatalyst. a) The FLP‐P‐Bi‐Co_3_O_4_ electrocatalyst consists of two moieties: FLP‐P nanosheets and Bi‐Co_3_O_4_ platelets. In the FLP‐P‐Bi‐Co_3_O_4_‐catalytic OER process, the cobalt (Co) center plays a crucial role in forming the Co‐OOH intermediate; meanwhile, the phosphate groups help to stabilize the reactive Co‐OOH. This dual functionality of the FLP‐P‐Bi‐Co_3_O_4_ electrocatalyst − enabling both formation and stabilization of the Co‐OOH intermediate − is called a bifunctional mechanism. (The details of this reaction mechanism are delineated in Figure 5c.) Subsequently, the secondary hydroxyl group of GLY coordinates selectively with the Bi center and is oxidized to DHA with the assistance of the Co‐OOH intermediate. b) A schematic illustration outlines the electrocatalytic upgrading process from GLY to DHA, by utilizing FLP‐P‐Bi‐Co_3_O_4_ electrodes for both anodic oxidation reactions and cathodic HER in neutral medium. At the anode, water molecules are oxidized on the Bi‐Co_3_O_4_ surface, leading to the formation of the Co‐OH and Co‐OOH intermediates as identified and optimized by in situ Raman spectroscopy. These intermediates play a crucial role in the GLY conversion to DHA. Simultaneously at the cathode, electrons collected from the anode via an external circuit facilitate the reduction of protons (H^+^) to generate H_2_.

In electrocatalytic water splitting, the oxygen evolution reaction (OER) consists of four consecutive proton‐coupled electron transfer steps via three surface‐bound intermediates of different oxidation abilities: *OH, *OOH, and *O (with * representing the active center bound on an electrocatalyst).^[^ ^18^, ^19^ ^]^ According to the earlier electrocatalysis studies, the oxidation of GLY is governed predominantly by the *OH intermediate, where the strong oxidation potential of *OH ensures an efficient oxidation of GLY, but its selective oxidation toward DHA is hampered by overoxidation.^[^ ^20^, ^21^ ^]^ On the other hand, the *OOH intermediate formed in the electro‐oxidation of GLY is considered responsible for its highly selective conversion to DHA.^[^ ^22^ ^]^ From an energy perspective, the formation of the catalyst‐bound OER intermediates (i.e., *OH, *OOH, and *O) increases the overpotential of a water splitting process, in which the generation of *OOH via the nucleophilic attack of a water molecule on *O is regarded as an energy demanding step.^[^ ^23^ ^]^ To address this issue, we recently applied a bifunctional mechanism (i.e., enabling both formation and stabilization of the reactive *OOH intermediate, as elucidated in **Figure** **1**a) to facilitate the electrocatalytic OER in neutral medium, where the energy demanding step of forming the *OOH intermediate is eased by the proton transfer from *OOH to a neighboring catalytic site to stabilize the *OOH formation.^[^ ^23^ ^−^ ^25^ ^]^

It should be emphasized that the formation of the OER intermediates in electrocatalytic reactions is potential dependent, i.e., the concentration of a particular intermediate can be maximized at a certain electrochemical potential.^[^ ^26^ ^]^ The successful utilization of this concept relies on the in situ detection of these intermediates to accurately determine the optimal conditions for each intermediate production. However, identifying the reaction intermediates in liquid environment is highly challenging due to the formation of these reactive species of low stabilities and short lifetimes.^[^ ^27^ ^]^ Until now, there is no report on the selective conversion of GLY to DHA based on the proper in situ spectroscopic identification of the OER intermediates. As a result, the employed electrocatalysts could suffer from unsatisfying catalytic performance.^[^ ^28^ ^]^

In this study, for using the OER intermediates to assist the GLY oxidation to DHA in neutral aqueous medium (Figure 1a), we herein synthesized an electrocatalyst by combining phosphated few‐layer phosphorene (FLP‐P) nanosheets with bismuth‐doped cobalt oxide (Bi‐Co_3_O_4_) platelets. In our synthesized FLP‐P‐Bi‐Co_3_O_4_ electrocatalyst, the spinel cobalt oxide (Co_3_O_4_) platelets stood out, among non‐noble metal‐based electrocatalysts, because of their controllable electronic structures with two mixed valence states (Co^2+^ and Co^3+^), rich redox chemistry, Earth abundance, and environmental friendliness.^[^ ^29^ ^]^ Prior research has shown that the Co^2+^ and Co^3+^ in the Co_3_O_4_ platelets play distinct roles in OER; while Co^3+^ tends to bind with the hydroxyl groups of a reactant molecule, Co^2+^ acts as the primary active site to significantly influence the OER activity.^[^ ^30^ ^]^ Therefore, an effective control of the Co^2+^/Co^3+^ ratio in spinel Co_3_O_4_ platelets (e.g., by tuning the elemental doping, or coupling with an electron‐rich noble metal) is recognized as a promising approach to manipulate their electronic structures for enhancing the OER performance.^[^ ^31^, ^32^ ^]^

Few‐layer phosphorene (FLP) nanosheets are a 2D semiconductor material of a tunable direct bandgap, unique anisotropic property, and high carrier mobility, which have attracted tremendous interest for serving as an electrocatalyst in OER.^[^ ^33^, ^34^ ^]^ Although FLP nanosheets in a long‐term electrocatalytic OER process may suffer from structural degradation due to the active lone‐pair electrons exposed on the FLP surface, this FLP degradation can be suppressed effectively in the FLP‐P‐Bi‐Co_3_O_4_ hybrid by the electron transfer from FLP‐P to the other Co_3_O_4_ moiety, because of the relatively higher Fermi level of the former (as depicted in Figure 3g). Upon the electron transfer, parts of the Co^3+^ ions are reduced to Co^2+^ in Co_3_O_4_, thus creating more active sites for OER and favorably enhancing the electrocatalytic activity.^[^ ^32^, ^35^ ^]^ In addition, the phosphate groups (denoted by P) modified on the FLP surface of FLP‐P‐Bi‐Co_3_O_4_ facilitate the OER efficiency via a bifunctional mechanism (Figure 1a),^[^ ^24^ ^]^ where the Co center promotes the formation of the cobalt‐oxo intermediates (i.e., Co‐OOH) and the neighboring phosphate groups help to stabilize the Co‐OOH intermediate through a proton transfer.

We also applied operando Raman spectroscopy to identify the key intermediates (i.e., Co‐OH and Co‐OOH) during OER in the FLP‐P‐Bi‐Co_3_O_4_‐assisted electrocatalytic water splitting, and confirmed the reaction mechanism based on experimental observations. Taking advantage of in situ Raman spectroscopic detections, we optimized the FLP‐P‐Bi‐Co_3_O_4_‐catalytic GOR by tuning electrochemical potential for the maximum production of the Co‐OOH intermediate. Our study reveals that the Bi species, doped in the FLP‐P‐Bi‐Co_3_O_4_ electrocatalyst, prefers to coordinate with the secondary hydroxyl group of GLY, enabling the selective oxidation of GLY to DHA. Consequently, an extraordinary electrocatalytic activity was achieved by successfully converting GLY (85% in conversion) to DHA (89% in selectivity). Furthermore, we couple this anodic GOR with the cathodic water electrolysis to achieve the simultaneous hydrogen production with high efficiency (Figure 1b). This work offers a practice guideline for the future design of employing the OER intermediates in an organic oxidation reaction system, in parallel with the hydrogen production, and provides both economic and environmental benefits, while addressing key challenges in resource utilization and efficiency.

## 2 Results and Discussion

### 2.1 Preparation and Characterization of Electrocatalysts

**Figure 2:**
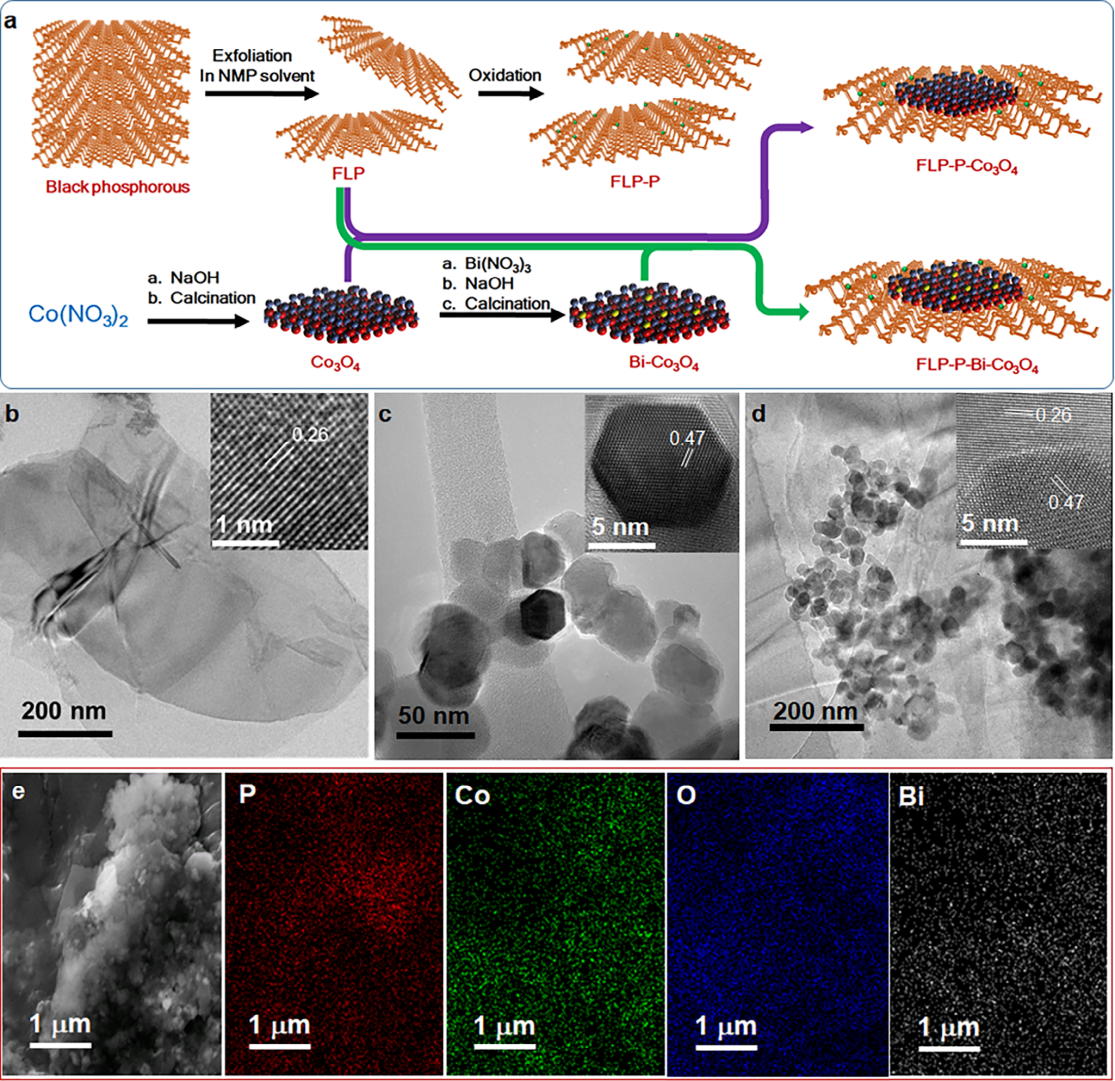
Material syntheses and electron microscopic characterizations. a) A schematic illustration is presented for the syntheses of different electrocatalysts. b−d) The TEM and HR‐TEM (in the insets) images of b) FLP‐P, c) Bi‐Co_3_O_4,_ and d) FLP‐P‐Bi‐Co_3_O_4_ reveal their crystalline structures. e) The SEM image of FLP‐P‐Bi‐Co_3_O_4_ together with the EDS elemental mappings (in the right panels) of phosphorous (P, red), cobalt (Co, green), oxygen (O, blue), and bismuth (Bi, white).

The electrocatalysts were synthesized via a simple solution‐based method as illustrated in **Figure** **2**a. Black phosphorus was exfoliated ultrasonically in N‐methyl‐2‐pyrrolidone (NMP) solvent to generate FLP nanosheets, followed by a controlled oxidation process to produce the phosphated FLP nanosheets (denoted by FLP‐P). Subsequently, cobalt and bismuth hydroxides were precipitated from a solution containing cobalt nitrate and bismuth nitrate, which were then calcined to form Bi‐Co_3_O_4_ platelets. The final product, FLP‐P‐Bi‐Co_3_O_4_, was obtained by sonicating the FLP‐P nanosheets with Bi‐Co_3_O_4_ platelets in NMP and stirring the mixture for 12 h. For later comparative analysis, pure Co_3_O_4_, FLP‐P‐Co_3_O_4_, and FLP‐Bi‐Co_3_O_4_ were also synthesized using a similar methodology. The details of experimental processes and instrumentation methods can be found in the supplemental information.

The transmission electron microscopy (TEM) images of FLP (Figure S2a, Supporting Information) and FLP‐P (Figure 2b) show a 2D sheet‐like structure of smooth, lamellar morphology with the same lattice spacing of 0.26 nm (in the insets of Figure S2a, Supporting Information; Figure 2b), corresponding to the (040) planes of black phosphorus.^[^ ^35^ ^]^ The TEM image in Figure S2b (Supporting Information) exhibits the hexagonal platelet morphology of Co_3_O_4_ with an interplanar spacing of 0.47 nm (in the inset of Figure S2b, Supporting Information) attributed to the (111) planes of spinel Co_3_O_4_.^[^ ^36^ ^]^ With the introduction of trace Bi to Co_3_O_4_ (represented as Bi‐Co_3_O_4_), variations of the hexagonal platelet morphology (Figure 2c) and the interplanar spacing (in the inset of Figure 2c) of Co_3_O_4_ are negligible. After the deposition of Bi‐Co_3_O_4_ onto FLP‐P, the TEM image in Figure 2d displays hexagonal Bi‐Co_3_O_4_ platelets dispersed over FLP‐P nanosheets, of which the lattice‐resolved high‐resolution transmission electron microscopy (HR‐TEM) image (in the inset of Figure 2d) shows the crystal planes of both Bi‐Co_3_O_4_ (111) and FLP‐P (040) distinctly in the composite structure. The FLP‐P nanosheets provide a large surface area, facilitating effective dispersion and interaction with the Bi‐Co_3_O_4_ platelets. The cooperation between these two moieties of FLP‐P‐Bi‐Co_3_O_4_ can lead to enhancing electronic interactions, charge transfer, and the overall catalytic activity. The TEM and HR‐TEM images of FLP‐Bi‐Co_3_O_4_ (Figure S3a, Supporting Information) and FLP‐P‐Co_3_O_4_ (Figure S3b, Supporting Information) resemble those of FLP‐P‐Bi‐Co_3_O_4_ (Figure 2d).

In Figure 2e, a large‐area scanning electron microscopy (SEM) image of FLP‐P‐Bi‐Co_3_O_4_ displays the overall morphology of this composite catalyst with the energy‐dispersive X‐ray spectroscopy (EDS) elemental mappings of the uniformly distributed phosphorus (P, red), cobalt (Co, green), oxygen (O, blue), and bismuth (Bi, white) in the entire area. This uniformity is crucial as it shows the effective integration of Bi‐Co_3_O_4_ and FLP‐P, potentially enhancing the electrocatalytic properties by ensuring that all components are well‐dispersed and accessible for reactions. For comparison, the SEM image of FLP‐P‐Co_3_O_4_ together with its EDS mappings (Figure S3c, Supporting Information) was also taken to demonstrate the presence and uniform distributions of phosphorous, cobalt, and oxygen.

**Figure 3:**
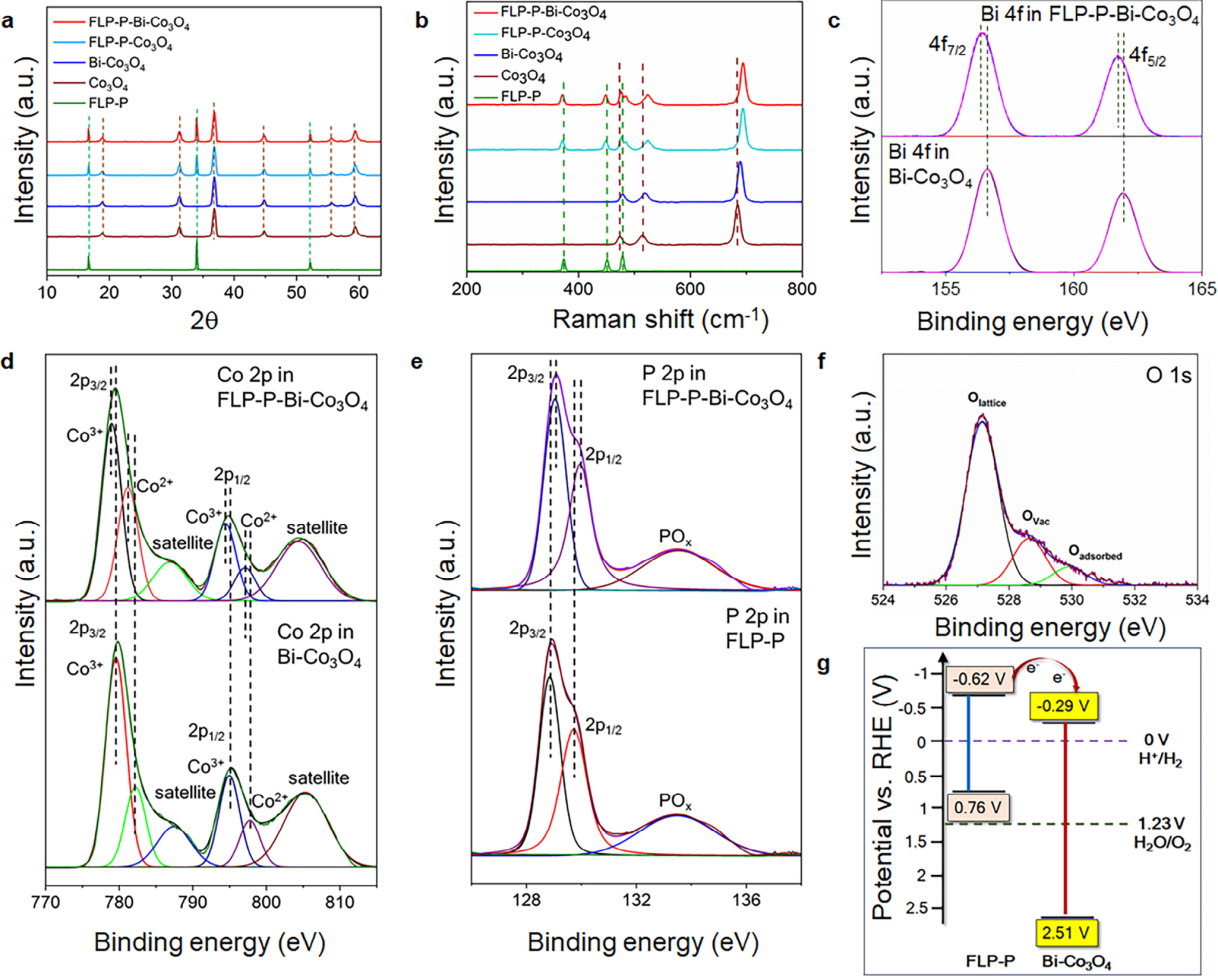
Spectroscopic characterizations and electronic interactions in the FLP‐P‐Bi‐Co_3_O_4_ electrocatalyst. a,b) The a) XRD and b) Raman scattering spectra of the as‐synthesized electrocatalysts were used to analyze their crystalline structures and vibrational characteristics. c−f) The binding energies of the c) Bi 4f, d) Co 2p, e) P 2p, and f) O 1s core levels were investigated by XPS for the Bi‐Co_3_O_4_, FLP‐P, and FLP‐P‐Bi‐Co_3_O_4_ electrocatalysts. g) The energy band diagram of the adjacent FLP‐P and Bi‐Co_3_O_4_ moieties inside the FLP‐P‐Bi‐Co3O4 hybrid electrocatalyst showing the electronic band structure and an interfacial electron transfer.

In **Figure** **3**a, both Co_3_O_4_ and Bi‐Co_3_O_4_ exhibit identical X‐ray diffraction (XRD) patterns with the peaks at 2*θ* values of 18.8°, 31.2°, 36.8°, 44.8°, 55.6°, and 59.4°, corresponding respectively to the (111), (220), (311), (400), (422), and (511) crystal planes of standard cubic spinel Co_3_O_4_ (JCPDS No. 65–3103).^[^ ^36^ ^]^ The absence of the diffraction peaks of the Bi species in the Bi‐Co_3_O_4_ platelets, such as metallic Bi or Bi_2_O_3_, indicates a high dispersion of the Bi dopant throughout the Bi‐Co_3_O_4_ crystal structure. Furthermore, the XRD spectrum of FLP‐P‐Co_3_O_4_ involves the signals of both FLP‐P and Co_3_O_4_; likewise, the XRD pattern of FLP‐P‐Bi‐Co_3_O_4_ contains those peaks of FLP‐P and Bi‐Co_3_O_4_, confirming that all constituents exist in the hybrid structure. The retention of characteristic peaks from both constituents of the composite catalyst suggests that the hybrid structure maintains the crystalline integrity of each material.

The interfacial electronic interactions between the constituents in composite materials were effectively characterized by combining Raman scattering spectroscopy and X‐ray photoelectron spectroscopy (XPS). The Raman spectra in Figure 3b display the prominent vibrational signals of FLP‐P, Co_3_O_4_, Bi‐Co_3_O_4_, FLP‐P‐Co_3_O_4_, and FLP‐P‐Bi‐Co_3_O_4_. For FLP‐P, the peaks centered at 372, 448, and 475 cm^−1^ are assigned to the *A*_g_^1^ (out‐of‐plane), *B*_2g_ (in‐plane), and *A*_g_^2^ (in‐plane) vibrational modes of black phosphorus, respectively.^[^ ^37^ ^]^ The Raman signals for Co_3_O_4_ are found at 485 cm^−1^ (*E*_g_), 524 cm^−1^ (*F*_2g_), and 694 cm^−1^ (*A*_1g_) with both *E*_g_ and *F*_2g_ characterizing the Co−O bending vibrations, while A_1g_ representing the Co−O stretching.^[^ ^16^, ^38^ ^]^ After the inclusion of Bi in Bi‐Co_3_O_4_, the vibrational modes are blue‐shifted, as compared with those of spinel Co_3_O_4_, because the large Bi^3+^ ions substitute the Co^3+^ ions to occupy the octahedral sites of Co_3_O_4_.^[^ ^39^, ^40^ ^]^ In FLP‐P‐Bi‐Co_3_O_4_, blue‐shifts of the Raman signals of Bi‐Co_3_O_4_ were observed, while red‐shifts were detected for those of FLP‐P. The same trend of the Raman signal shifts was also observed for the FLP‐P‐Co_3_O_4_ hybrid.

The electronic states and interactions of the surface elemental configurations for different electrocatalysts were further explored by X‐ray photoelectron spectroscopy (XPS). In Figure 3c, the Bi 4f core level XPS spectrum of FLP‐P‐Bi‐Co_3_O_4_ shows the strong doublet signals at 156.4 and 161.7 eV, corresponding to Bi 4f_7/2_ and Bi 4f_5/2_, respectively. There is no trace of the metallic Bi^0^, Bi^2+^, and Bi^5+^ 4f core level signals, revealing that only the Bi^3+^ state is present in FLP‐P‐Bi‐Co_3_O_4_. However, compared with Bi‐Co_3_O_4_, the binding energies of Bi 4f in FLP‐P‐Bi‐Co_3_O_4_ shift to lower values. In the Co 2p XPS spectra of FLP‐P‐Bi‐Co_3_O_4_ and Bi‐Co_3_O_4_ (Figure 3d), the peaks centered at 779.2 (2p_3/2_) and 794.1 (2p_1/2_) eV confirm the existence of the Co^3+^ species, while the signals detected at 781.4 eV (2p_3/2_) and 796.2 (2p_1/2_) eV reveal the presence of Co^2+^. The red‐shifts of the Co 2p signals of Bi‐Co_3_O_4_, relative to those of FLP‐P‐Bi‐Co_3_O_4_, are marked by the dashed lines. Additionally, the remaining two broad peaks observed at the binding energies of ≈787.1 and ≈803.9 eV are ascribed to the satellite peaks.^[^ ^41^ ^]^ In the high‐resolution P 2p XPS spectrum of FLP‐P, two intense peaks centered at 129.1 and 130.0 eV are attributed respectively to the 2p_3/2_ and 2p_1/2_ of a P–P bond, while a broad peak at ≈133.0 eV is due to the oxidation of FLP‐P.^[^ ^42^ ^]^ Interestingly, the P 2p peaks of FLP‐P exhibit blue‐shifts to the higher binding energies (indicated by the dash lines) after FLP‐P was coupled with Bi‐Co_3_O_4_ in FLP‐P‐Bi‐Co_3_O_4_.

The XPS analysis reveals a positive shift in the binding energy of the P 2p core level, indicating an increase in the oxidation state of phosphorus due to the electron depletion. Conversely, the Bi 2p and Co 2p core levels both exhibit a negative shift in binding energy, consistent with the electron accumulation. These observations suggest a net electron migration from FLP‐P to Bi‐Co_3_O_4_ at the interface. This charge transfer is further supported by Raman spectroscopy. The characteristic vibrational modes of FLP‐P exhibit a red‐shift, implying phonon softening associated with the reduced electron density. In contrast, the Bi‐Co_3_O_4_ vibrational bands display a blue‐shift, indicative of phonon hardening due to the electron enrichment. The complementary nature of the XPS and Raman spectral analyses provides a compelling evidence for the interfacial electron migration from FLP‐P to Bi‐Co_3_O_4_, which is expected to modulate electronic structure and enhance charge separation within the composite.

To support the oxidation of FLP to FLP‐P, the P 2p core level XPS and FTIR spectroscopic investigations were conducted. In FLP (prepared from freshly exfoliated BP), the P 2p_3/2_ and P 2p_1/2_ peaks appear at 129.1 and 130.0 eV, respectively (Figure S4a, Supporting Information), with a weak oxide peak at ≈133.5 eV (in the inset of Figure S4a, Supporting Information), which is a typical unavoidable oxide peak for liquid exfoliated BP. Upon oxidation to form FLP‐P, a broad intense band at ≈133.5 eV emerges alongside the original peaks, indicating the presence of the oxidized phosphorus species (PO_x_), as shown in Figures 3e and S4a (Supporting Information). The broad FWHM reflects a range of the P─O and P═O bonding environments. The functionalization of phosphate groups in FLP‐P is further confirmed by FTIR with the P─O stretching vibrations at 1022 and 1176 cm^−1^ and the P═O stretching vibrations at 1391 and 1648 cm^−1^ (Figure S4b, Supporting Information).^[^ ^43^ ^]^ Furthermore, these peaks are weak in pure FLP, indicating its minimal oxidation.

In Figure 3f, the oxygen bonded in Co−O, the surface dangling bond (O⋅), and the adsorbed oxygen (O_2_ and/or H_2_O) of FLP‐P‐Bi‐Co_3_O_4_ are responsible for the O 1s core level peaks at 527.1, 528.6, and 529.8 eV, respectively. To elucidate the charge transfer between the FLP‐P and Bi‐Co_3_O_4_ moieties in the FLP‐P‐Bi‐Co_3_O_4_ hybrid, an energy band diagram (Figure 3g) was constructed, based on the UV–vis diffuse reflectance spectra (Figure S5, Supporting Information) and Mott‐Schottky plots (Figure S6, Supporting Information) for both FLP‐P and Bi‐Co_3_O_4_.^[^ ^44^, ^45^ ^]^ From the energy level diagram, an electron transfer from FLP‐P to Bi‐Co_3_O_4_ is suggested.

### 2.2 Electrochemical Activity and Catalytic Durability

**Figure 4:**
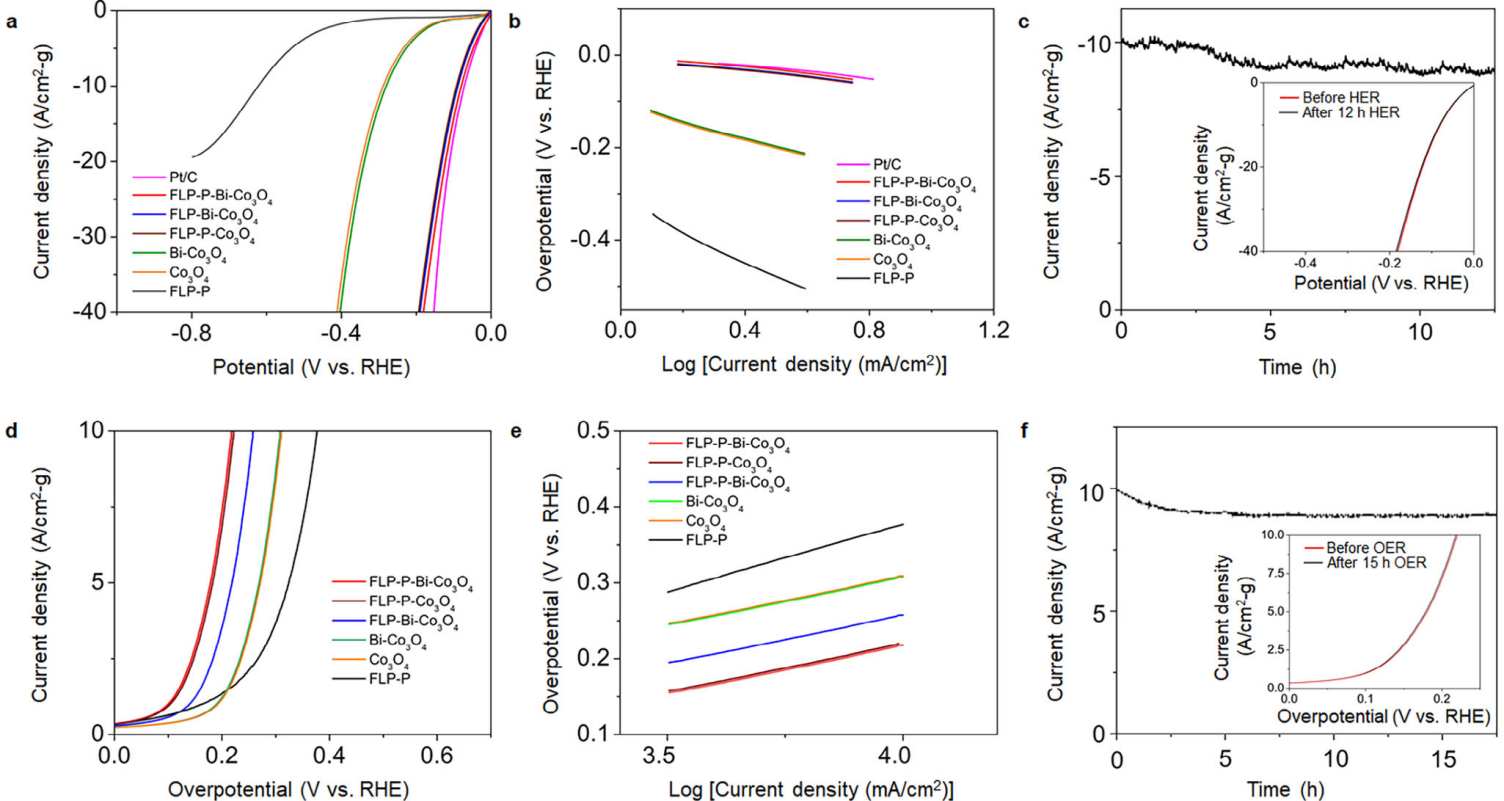
The HER and OER activities of various electrocatalysts. a) The polarization curves of HER were measured at a scan rate of 10 mV·s^−1^ using various electrocatalyst materials deposited on carbon cloth as the working electrodes in 0.5 m Na_2_SO_4_. b) The Tafel plots are derived from the HER polarization curves of (a). c) Long‐term stability of the FLP‐P‐Bi‐Co_3_O_4_ electrocatalyst during HER. The inset shows the polarization curves of HER before and after a 12 h reaction. d) The polarization curves of OER were scanned at 10 mV·s^−1^ using different electrocatalyst materials deposited on carbon cloth as the working electrodes in 0.5 m Na_2_SO_4_. e) The Tafel plots are derived from the OER polarization curves of (d). f) Long‐term stability of the FLP‐P‐Bi‐Co_3_O_4_ electrocatalyst during OER. The inset shows the polarization curves of OER before and after a 15 h reaction.

The electrocatalytic activities of different electrocatalysts in both HER and OER were evaluated using a three‐electrode system in 0.5 m Na_2_SO_4_. As shown in the linear sweep voltammetry (LSV) curves (**Figure** **4**a), FLP‐P‐Bi‐Co_3_O_4_ demonstrates an impressive catalytic activity for HER by achieving an overpotential of 88 mV to reach the current density of 10 A·cm^−2^·g^−1^, which is comparable to that (84 mV) of a commercial Pt/C catalyst. Notably, the as‐prepared electrocatalyst without phosphate functionalization (i.e., FLP‐Bi‐Co_3_O_4_) or bismuth doping (i.e., FLP‐P‐Co_3_O_4_) exhibits the similar HER activity to FLP‐P‐Bi‐Co_3_O_4_, indicating that these modifications do not significantly affect the HER performance.

The exceptional HER activity of FLP‐P‐Bi‐Co_3_O_4_ is further supported by the Tafel analysis (Figure 4b). The Tafel slope of 48 mV·dec^−1^ for FLP‐P‐Bi‐Co_3_O_4_ is comparable to 44 mV·dec^−1^ for Pt/C, suggesting that FLP‐P‐Bi‐Co_3_O_4_ operates with a similar mechanism to that of the Pt‐based catalysts, known for their efficiency of facilitating HER. This comparison highlights the potential of FLP‐P‐Bi‐Co_3_O_4_ to be used as a viable alternative to noble metal catalysts in the hydrogen production applications. In Figure 4c, the FLP‐P‐Bi‐Co_3_O_4_ electrocatalyst demonstrates an excellent electrochemical stability by retaining 96% of its initial current density over 12 h at −88 mV versus RHE. The cathodic current of HER (in the inset of Figure 4c) shows an unchanged LSV curve with minimal activity loss, confirming its high durability and suitability for long‐term HER applications. As compared in Table S1 (Supporting Information), the HER performance in neutral medium with an FLP‐P‐Bi‐Co_3_O_4_ electrode outperforms other recently reported electrocatalysts.

### 2.3 The OER Activity

In Figure 4d, FLP‐P‐Bi‐Co_3_O_4_ demonstrates its remarkable performance in OER, by achieving an overpotential of 166 mV to reach the current density of 10 A·cm^−2^·g^−1^. This performance of FLP‐P‐Bi‐Co_3_O_4_ is notably better than the individual FLP‐P and Bi‐Co_3_O_4_ electrodes with their overpotentials of 428 and 340 mV, respectively. As compared with FLP‐P‐Bi‐Co_3_O_4_, the absence of Bi dopants in FLP‐P‐Co_3_O_4_ does not significantly alter its OER efficiency. However, the removal of the phosphate functionalization (i.e., FLP‐Bi‐Co_3_O_4_) leads to a marked increase in overpotential by 39 mV, indicating the importance of these phosphate groups in enhancing the catalytic activity.

To further understand the kinetic characteristics of these electrocatalysts, the Tafel slopes (Figure 4e) derived from the OER polarization curves were analyzed. While the Tafel slopes of 56 and 58 mV·dec^−1^ were obtained for FLP‐P‐Bi‐Co_3_O_4_ and FLP‐P‐Co_3_O_4_, respectively, 88 mV·dec^−1^ was deduced for FLP‐Bi‐Co_3_O_4_. By contrast, FLP‐P and Bi‐Co_3_O_4_ hold the much higher Tafel slopes of 303 and 210 mV·dec^−1^, respectively. These results suggest that the FLP‐P‐Bi‐Co_3_O_4_ hybrid expedited faster reaction kinetics and higher electron transfer rates in comparison with its individual constituents.

The long‐term stability of FLP‐P‐Bi‐Co_3_O_4_ was assessed at a constant cell potential of 1.4 V over a period of 15 h. As shown in Figure 4f, the cell successfully maintained 98% of its initial current density after 15 h of continuous operation. Additionally, the anodic current for OER, displayed in the inset of the Figure 4f, exhibits an unchanged LSV curve with only a minor reduction in the catalytic activity. These outcomes demonstrate the impressive durability of the electrocatalyst material used in the OER applications, highlighting its robust performance over an extended period. As presented in Table S2 (Supporting Information), the OER performance in neutral medium with an FLP‐P‐Bi‐Co_3_O_4_ electrode is superior to other recently reported electrocatalysts.

### 2.4 Phosphate Functionalization

Further explorations on the enhanced OER catalytic activity of FLP‐P‐Bi‐Co_3_O_4_ over FLP‐Bi‐Co_3_O_4_ were performed to determine whether the reaction mechanism is relevant to the phosphate functionalization. Since the Co species of both FLP‐P‐Bi‐Co_3_O_4_ and FLP‐Bi‐Co_3_O_4_ electrocatalysts were generally regarded as the active catalytic centers for OER,^[^ ^46^ ^]^ we employed XPS to examine the oxidation states of Co in these catalysts. The Co 2p XPS spectra in Figure S7 (Supporting Information) show no significant change in the oxidation states of Co in these two electrocatalysts different by the phosphate functionalization.

Alternatively, the improved OER activity of FLP‐P‐Bi‐Co_3_O_4_ over FLP‐Bi‐Co_3_O_4_ could stem from an increase of the surface area due to phosphate functionalization. To this end, we compared the specific activities of both FLP‐P‐Bi‐Co_3_O_4_ and FLP‐Bi‐Co_3_O_4_ electrodes with the Brunauer–Emmett–Teller (BET) method (Figure S8, Supporting Information) to measure their electrochemical active surface areas (ECSA, Figure S9, Supporting Information). In view of the LSV curves normalized to the geometric surface areas measured by BET and ECSA (Figure S10, Supporting Information), no significant difference was observed in the OER efficiency by using the FLP‐P‐Bi‐Co_3_O_4_ or FLP‐Bi‐Co_3_O_4_ electrode, revealing that an increase of the surface area by phosphate functionalization is minimal. Furthermore, we investigated the impact of phosphate functionalization on electrical conductivity by electrochemical impedance spectroscopy (EIS). As demonstrated in Figure S11 (Supporting Information), no improvement in the electrical conductivity is found for FLP‐P‐Bi‐Co_3_O_4_.

**Figure 5:**
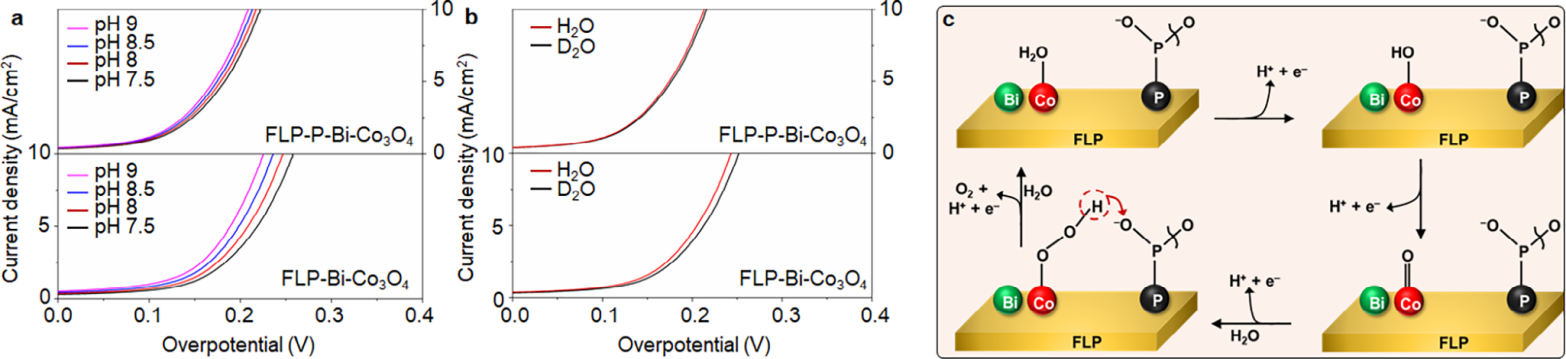
Effect of phosphate functionalization on the OER activity. a,b) The OER activities and proton‐diffusion kinetics at the electrolyte/catalyst interface are compared between the FLP‐P‐Bi‐Co_3_O_4_ and FLP‐Bi‐Co_3_O_4_ electrocatalysts through the LSV measurements a) at different pH (7.5−9) environments and b) with 0.5 m Na_2_SO_4_ dissolved in H_2_O, or D_2_O, as an electrolyte. c) An illustration of the phosphate‐assisted bifunctional mechanism. The reaction begins with the oxidation of a water molecule on the Co center of FLP‐P‐Bi‐Co_3_O_4_ to eventually form the Co‐OOH intermediate, which is stabilized through a proton transfer by the neighboring phosphate groups modified on the FLP surface.

The OER on the surface of a metal oxide catalyst has long been assumed to proceed via four successive proton‐coupled electron transfer (PCET) steps. In this metal oxide‐assisted electrocatalytic reaction, the surface metal cations (M^n+^) serve as active sites and considerable OER activity improves under highly basic conditions, but deteriorates in near‐neutral environments.^[^ ^47^ ^]^ Therefore, considering the rate‐determining step of de‐protonating Co‐OOH to Co‐OO* in the FLP‐P‐Bi‐Co_3_O_4_‐catalytic OER process, a change of the concentration of hydroxide ions (OH^−^) toward near‐neutral pH levels should lead to a significant decrease in the OER activity, if the proton transfer is with the help of OH^−^ ions in the electrolytic solution.^[^ ^48^ ^]^ We tested electrocatalytic activities of the FLP‐Bi‐Co_3_O_4_‐ and FLP‐P‐Bi‐Co_3_O_4_‐catalytic OER in different pH environments. By varying pH from 7.5 to 9 (**Figure** **5**a), the OER efficiency alters notably in the FLP‐Bi‐Co_3_O_4_‐catalytic reactions, in sharp contrast to the negligible change when using FLP‐P‐Bi‐Co_3_O_4_ as an electrocatalyst. These results suggest that the phosphate groups of FLP‐P‐Bi‐Co_3_O_4_ have engaged in the de‐protonation process and, consequently, changing the environmental pH value has little impact on the FLP‐P‐Bi‐Co_3_O_4_‐catalytic OER efficiency.

To explore the proton transfer mechanism, we further conducted an isotope‐labeling experiment by using D_2_O or H_2_O as an electrolyte solvent to analyze the related OER kinetics. In electrocatalytic reactions, the mobility of deuterons (D^+^) is slower than that of protons (H^+^), suggesting that utilizing a D_2_O solution could effectively slow down the OER kinetics, if the proton transfer is by way of the surrounding solution. When employing FLP‐P‐Bi‐Co_3_O_4_ or FLP‐Bi‐Co_3_O_4_ as an electrocatalyst, the electrochemical results presented in Figure 5b provide a comparison of the OER activities in H_2_O and D_2_O. The reactions assisted by FLP‐Bi‐Co_3_O_4_ demonstrate a pronounced H/D dependence, with a significant reduction of the OER current in D_2_O. By contrast, the FLP‐P‐Bi‐Co_3_O_4_‐catalytic reactions exhibit a minimal H/D dependence. Similar to earlier studies,^[^ ^24^, ^49^ ^]^ we attribute the enhanced OER activity of FLP‐P‐Bi‐Co_3_O_4_ over FLP‐Bi‐Co_3_O_4_ to the improved proton transfer kinetics. This accelerated proton transfer in FLP‐P‐Bi‐Co_3_O_4_ can be explained with a bifunctional mechanism (Figure 5c), where the phosphate groups act as a proton acceptor to facilitate the proton transfer and, consequently, improve the electrocatalytic OER activity.

### 2.5 In Situ Raman Spectroscopic Identification of the OER‐Intermediates

**Figure 6:**
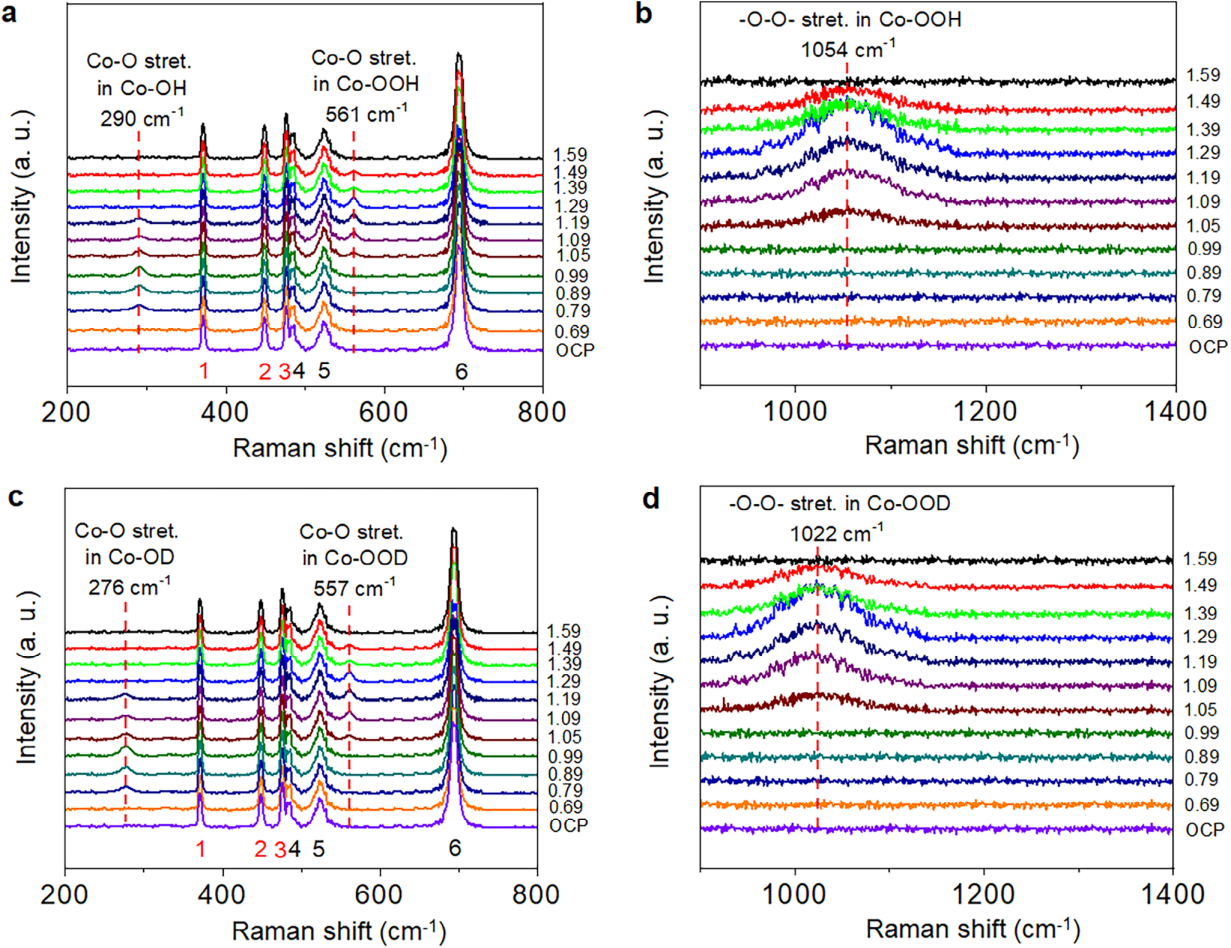
In situ Raman spectroscopic observation of the OER intermediates. a,b) In situ Raman spectra were recorded for the FLP‐P‐Bi‐Co_3_O_4_‐assisted electrocatalytic OER with 0.5 m Na_2_SO_4_ dissolved in AcCN:H_2_O (5:1) as an electrolyte. The spectra were scanned in the range of 200–1400 cm^−1^ as the electrochemical potential was adjusted from 0.69 to 1.59 V (vs RHE) with an incremental step of 0.1 V. While the peak at 290 cm^−1^ corresponds to the Co−O stretching vibration of the Co─OH intermediate, the signals at 561 and 1054 cm^−1^ are due to the Co−O and ‐O─O‐ stretching vibrations of the Co─OOH intermediate, respectively. c,d) The H/D isotope effect was examined by scanning the Raman spectra with the same experimental conditions as those of (a,b), except that 0.5 m Na_2_SO_4_ was dissolved in AcCN:D_2_O (5:1).

To detect the oxygen‐containing intermediates formed on the electrocatalyst surface in the FLP‐P‐Bi‐Co_3_O_4_‐assisted OER, in situ Raman spectroscopy was employed under a sequential increase of electrochemical potential. However, spectroscopic detection of the OER intermediates in water is highly challenging due to their extremely low steady‐state concentrations and short lifetimes. In our recent in situ Raman spectroscopic identification of the OER intermediates,^[^ ^50^ ^]^ we addressed this problem by using a mixture of acetonitrile:water = 5:1 (represented as AcCN:H_2_O (5:1)), instead of pure water, as an electrolyte to slow down the OER kinetics for successfully detecting the reactive intermediates. In **Figure** **6**a, at the open‐circuit potential (OCP), the signals detected at 372, 448, 475, 485, 524, and 694 cm^−1^ (as labeled by 1, 2, 3, 4, 5, and 6, respectively) are in accordance with the typical Raman‐active modes of the FLP‐P‐Bi‐Co_3_O_4_ electrocatalyst observed by ex situ Raman spectroscopy (i.e., the same Raman signals as shown in Figure 3b).

When the potential increases to 0.79 V, a new Raman signal emerges at ≈290 cm^−1^ and becomes stronger as the potential increases. This signal reaches a maximum intensity at 0.99 V, then decreases, and finally disappears at 1.29 V. According to previous studies, the Raman signal at ≈290 cm^−1^ is attributed to the Co−O stretching vibration (*E*_g_) of the Co─OH intermediate,^[^ ^51^ ^]^ which was formed on the near‐surface Co center. While the signal at ≈290 cm^−1^ starts to decrease, a new peak at ≈561 cm^−1^ appears at 1.05 V. The intensity of this new peak increases gradually, reaches a maximum at 1.29 V, and disappears above 1.49 V. Based on previous reports,^[^ ^29^ ^]^ the peak at ≈561 cm^−1^ is assigned to the Co−O stretching vibration of the Co‐OOH intermediate. To support the existence of Co‐OOH, we further searched for the peroxide stretching vibration (denoted by ‐O−O‐) of this Co‐OOH intermediate. In Figure 6b, the signal at ≈1054 cm^−1^ shows up simultaneously with the Co−O stretching peak of Co‐OOH (at ≈561 cm^−1^ in Figure 6a) in the potential range of 1.05−1.49 V, suggesting that these two signals originate from the same chemical species. On this basis, the peak at ≈1054 cm^−1^ is ascribed to the ‐O−O‐ stretching vibration of Co‐OOH, which is not only in accordance with the previous assignment,^[^ ^29^ ^]^ but also validates the presence of the Co‐OOH intermediate.

Furthermore, we performed the H/D isotope‐labeling experiment to support the in situ Raman spectroscopic identification of the Co‐OH and Co‐OOH intermediates. As compared in Figure 6a,c, a red‐shift of the Co−O stretching from 290 to 276 cm^−1^ was observed after Co‐OH was deuterated to Co‐OD. In contrast to the vibrational signals of Co‐OOD (Figure 6c,d), both Co−O and ‐O−O‐ stretching vibrations of Co‐OOH (Figure 6a,b) are red‐shifted by 4 cm^−1^ (from 561 to 557 cm^−1^) and 32 cm^−1^ (from 1054 to 1022 cm^−1^), respectively, where the more prominent red‐shift in the stretching of ‐O−O‐, than Co−O, results obviously from the adjacency of the deuterium (D) to the vibrating site. From the spectroscopic investigation of Figure 6a,b, the maximal production of the Co‐OOH intermediate in the FLP‐P‐Bi‐Co_3_O_4_‐catalytic OER was found to occur at the electrochemical potential of 1.29 V. In the following FLP‐P‐Bi‐Co_3_O_4_‐assisted electrocatalytic biomass upgrading experiments, we fixed the potential at 1.29 V to gain the Co‐OOH intermediate for oxidizing GLY to DHA.

### 2.6 The GOR Activity

To investigate the electrocatalytic GOR activities of various electrocatalysts, a series of electrochemical measurements were conducted using a three‐electrode system with 0.5 m Na_2_SO_4_ containing 0.1 m GLY as an electrolyte. The primary competing reaction against GOR in this experimental condition is OER that could occur during water oxidation. Ideally, an electrocatalyst of GOR should exhibit a preference for GOR, while demonstrating minimal catalytic activity of OER. For comparison, the electrocatalytic activity and product selectivity toward OER through the use of these electrocatalysts were also examined under the same experimental condition of proceeding GOR, but in the absence of GLY.

**Figure 7:**
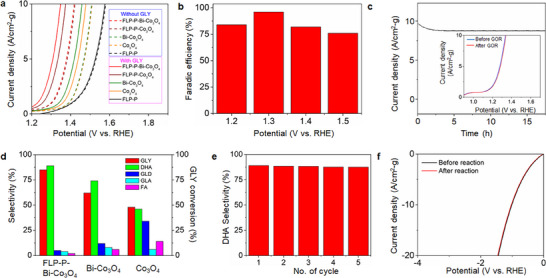
Performance merits of the FLP‐P‐Bi‐Co_3_O_4_ electrocatalyst in GOR. a) The OER (without GLY) and GOR (with GLY) polarization curves scanned at 20 mV·s^−1^ are compared for different electrocatalysts‐assisted GLY oxidation reactions in 0.5 m Na_2_SO_4_ solution. b) The Faraday efficiencies of the FLP‐P‐Bi‐Co_3_O_4_‐catalytic GLY conversion to DHA are presented with various applied potentials. c) Long‐term stability of the FLP‐P‐Bi‐Co_3_O_4_ electrocatalyst during GOR. The inset shows the polarization curves of GOR before and after a 15 h reaction. d) Product distribution of the GLY conversion reactions using different anode electrocatalysts. e) The conversion efficiency of the GLY oxidation to DHA using an FLP‐P‐Bi‐Co_3_O_4_ electrode for five consecutive experimental cycles. f) The LSV curves of the FLP‐P‐Bi‐Co_3_O_4_‐catalytic HER were measured at the scan rate of 10 mV s^−1^ in 0.5 m Na_2_SO_4_ solution.

**Figure** **7**a shows the LSV curves (based on the geometric current densities, mA cm^−2^) of different electrocatalysts with or without the presence of GLY. Using the FLP‐P electrocatalyst, the potential for OER (1.60 V) at the current density of 10 mA cm^−2^ shifts lower slightly after adding 0.1 m GLY, indicating its poor selectivity for GOR. In the absence of GLY, the Co_3_O_4_ or Bi‐Co_3_O_4_ electrocatalyst drives OER with very similar overall electrode performances. The addition of 0.1 m GLY significantly increases the current density for both Co_3_O_4_ and Bi‐Co_3_O_4_, demonstrating their preferential GOR over OER. Notably, applying Bi‐Co_3_O_4_ exhibits a lower anodic potential (1.52 V) than using Co_3_O_4_ (1.48 V) by 40 mV at the current density of 10 mA cm^−2^, indicating that the Bi doping enhances the GOR activity. Compared with the FLP‐P, Bi‐Co_3_O_4_, and FLP‐P‐Co_3_O_4_ electrocatalysts, FLP‐P‐Bi‐Co_3_O_4_ holds a lower potential of ≈1.3 V after introducing GLY, revealing that the GOR activity can be improved by the Bi doping, and/or the hybrid electrocatalyst.

Electrochemical potential represents a pivotal parameter to electrocatalytic GOR. To determine the Faraday efficiency (*FE*) of an electrocatalytic GOR to DHA, we tested the FLP‐P‐Bi‐Co_3_O_4_ electrode at four different electrochemical potentials. As presented in Figure 7b, the FLP‐P‐Bi‐Co_3_O_4_‐assisted GOR exhibits its *FE* > 90% at 1.3 V. This finding accords with the maximum production of Co‐OOH at an applied potential of 1.29 V observed by in situ Raman spectroscopy (Figure 6a,b), and further supports the role of the Co‐OOH intermediate in facilitating GOR. Demonstrated in Figure 7c is the long‐term electrocatalytic stability of the FLP‐P‐Bi‐Co_3_O_4_‐assisted GOR at 1.29 V. The products of catalysts‐assisted GLY oxidations were analyzed by high‐performance liquid chromatography (HPLC) with the calibrated curves of the standard GOR products shown in Figure S12 (Supporting Information). A representative chromatogram of the GOR products obtained after a 1.5 h reaction (Figure 7d; Table S3, Supporting Information), including the maximum production of DHA together with the other yields of glyceraldehyde (GLD), glyceric acid (GLA), and formic acid (FA), is presented in Figure S13 (Supporting Information). The FLP‐P‐Bi‐Co_3_O_4_‐assisted GOR was performed at 1.29 V with the electrochemical potential optimized from in situ Raman spectroscopic experiments, where the water oxidation was performed in the electrolyte of 0.5 m Na_2_SO_4_ dissolved in the AcCN:H_2_O (5:1) solvent. However, the GOR was conducted in the aqueous electrolyte of 0.5 m Na_2_SO_4_ containing 0.1 m GLY. To examine the influence of the AcCN:H_2_O (5:1) solvent in the product distribution of GOR, we performed the GOR measurements in both conditions (i.e., AcCN:H_2_O (5:1) versus pure H_2_O) at 1.29 V. Figure S14 (Supporting Information) shows that the GLY conversion in AcCN:H_2_O (5:1) is less than that in the aqueous electrolyte; however, the product distribution of GOR is the same by employing either solvent. Therefore, we conclude that using the AcCN:H_2_O (5:1) solvent in the in situ Raman experiments reduces the reaction rate of OER, which results in the reduced GLY conversion, but without altering the reaction pathways. The FLP‐P‐Bi‐Co_3_O_4_ electrode also shows excellent electrocatalytic durability with the negligible loss of efficiency after five reaction cycles (Figure 7e). As displayed in Figure S15 (Supporting Information), the Raman spectra of an FLP‐P‐Bi‐Co_3_O_4_ electrode are almost the same before and after it was used in GOR for five reaction cycles, indicating no significant change of the material properties throughout the reaction. In Table S4 (Supporting Information), the selective conversion of GLY to DHA in the FLP‐P‐Bi‐Co_3_O_4_‐assisted GOR exhibits a higher efficiency than those with other recently reported electrocatalysts.

To test the efficiency of an FLP‐P‐Bi‐Co_3_O_4_ electrocatalyst used in the electrochemical oxidation of GLY alongside the hydrogen generation, a neutral electrolyzer was constructed with the FLP‐P‐Bi‐Co_3_O_4_ electrode serving as both anode and cathode (as illustrated in Figure 1b). As shown in Figure S16 (Supporting Information), a cell voltage of just 1.37 V is sufficient to achieve the current density of 10 mA·cm^−2^. A long‐term stability test of the FLP‐P‐Bi‐Co_3_O_4_ electrolyzer was also conducted at a constant potential of 1.37 V over a duration of 10 h to evaluate the electrocatalytic performance. The results shown in Figure 7f indicate that the electrolyzer consistently maintained its current output, demonstrating that the efficiency of anodic GLY conversion was not adversely affected by the simultaneous cathodic hydrogen production, as evidenced by the unchanged polarization curves.

**Figure 8:**
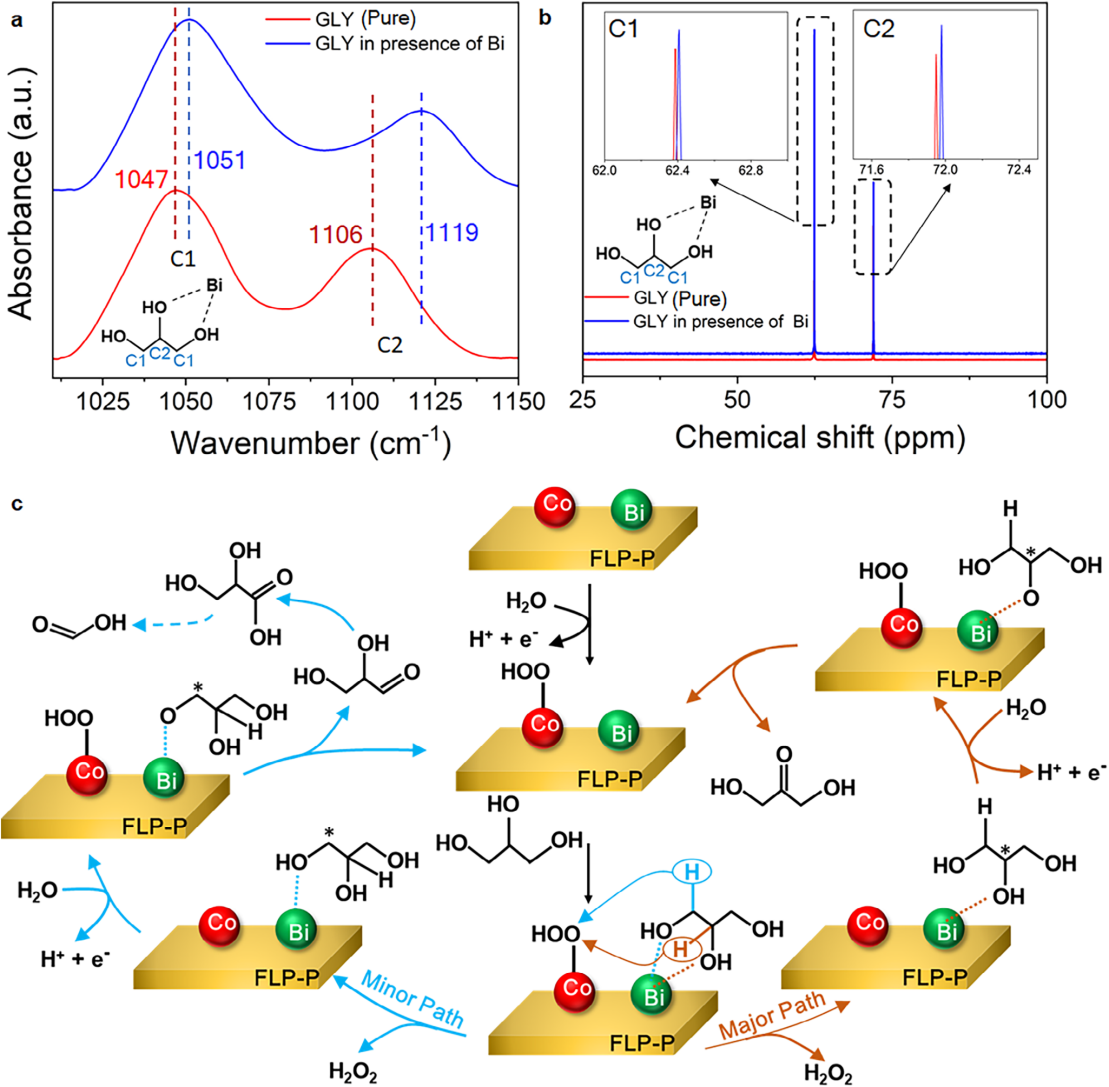
Spectroscopic tests for a proposed reaction mechanism in the FLP‐P‐Bi‐Co_3_O_4_‐assisted selective GLY oxidation to DHA. a) The FTIR investigation of pure GLY as against GLY in the presence of the FLP‐P‐Co_3_O_4_ or FLP‐P‐Bi‐Co_3_O_4_ electrocatalyst. b) The ^13^C NMR analysis for pure GLY as against GLY in the presence of the FLP‐P‐Co_3_O_4_ or FLP‐P‐Bi‐Co_3_O_4_ electrocatalyst. c) A proposed reaction mechanism for the selective oxidation of GLY to DHA in the FLP‐P‐Bi‐Co_3_O_4_‐assisted GOR.

From the experimental results with different electrocatalysts (Figure 7a), the addition of Bi to an electrocatalyst can significantly improve the GLY oxidation with high DHA selectivity. The adsorption configuration of reactant molecules on the catalyst surface is crucial for the reaction route and product selectivity. We applied Fourier‐transform infrared (FTIR) spectroscopy to investigate the interfacial association of GLY with different electrocatalysts. The characteristic bands of pure GLY observed at 1042 and 1112 cm^−1^ (**Figure** **8**a, the red trace) correspond to the stretching vibrations of primary (1°, C1) and secondary (2°, C2) alcohols, respectively. In the presence of FLP‐P, the positions of these vibrational bands remain unchanged (Figure S17, Supporting Information), indicating no significant interaction between GLY and FLP‐P. The very similar spectra for the coordination of GLY with Co_3_O_4_ or FLP‐P‐Co_3_O_4_ (Figure S18, Supporting Information), and for the association of GLY with Bi‐Co_3_O_4_ or FLP‐P‐Bi‐Co_3_O_4_ (Figure S19, Supporting Information), further reveal that FLP‐P does not affect the interaction of GLY with the electrocatalysts. However, after FLP‐P‐Co_3_O_4_ is introduced (Figure S18, Supporting Information, the green trace), the C1 peak of GLY shifts from 1042 to 1051 cm^−1^, while the C2 peak remains relatively unchanged, indicating that GLY coordinates with the Co center of the electrocatalyst mainly through the primary hydroxyl group. In contrast, after FLP‐P‐Bi‐Co_3_O_4_ is added (Figure S19, Supporting Information, the blue trace), the C2 peak of GLY shifts significantly from 1112 to 1123 cm^−1^ (also compared with no shift of the C2 peak of GLY when Bi is absent in FLP‐P‐Co_3_O_4_, Figure S18, Supporting Information), while the C1 peak shifts from 1042 to 1049 cm^−1^. This outcome suggests that the Bi center of the FLP‐P‐Bi‐Co_3_O_4_ electrocatalyst binds with both primary and secondary hydroxyl groups of GLY, but with the latter much stronger than the former.

We further performed the ^13^C NMR investigation to elucidate the binding interaction of GLY with different electrocatalysts. The ^13^C NMR spectrum of pure GLY (Figure 8b, the red trace) shows the characteristic peaks at 62.38 and 71.94 ppm, corresponding to the C1 and C2 carbons, respectively. The electron‐withdrawing effect due to the electronegative oxygen atom in the hydroxyl groups of GLY de‐shields the oxygen‐attached carbon nuclei, causing them to resonate at higher ppm values. In the presence of FLP‐P, the NMR peaks of GLY remain unchanged (Figure S20, Supporting Information), suggesting no significant interaction between FLP‐P and GLY. Moreover, the NMR spectra of GLY are identical when GLY is in the presence of Co_3_O_4_ or FLP‐P‐Co_3_O_4_ (Figure S21, Supporting Information), and also when GLY associates with Bi‐Co_3_O_4_ or FLP‐P‐Bi‐Co_3_O_4_ (Figure S22, Supporting Information). These results further indicate that FLP‐P does not affect the coordination of GLY with the electrocatalysts. However, relative to the spectrum of pure GLY, when GLY coordinates with FLP‐P‐Co_3_O_4_, the C1 peak shifts to a higher value (62.42 ppm), while the C2 peak remains largely unchanged (at 71.95 ppm, Figure S21, Supporting Information, the green trace), suggesting that the Co center in the electrocatalyst interacts with GLY via the primary hydroxyl groups. In contrast, when GLY associates with FLP‐P‐Bi‐Co_3_O_4_ (Figure 8b, the blue trace), both the C1 and C2 peaks shift to higher ppm values (of 62.41 and 71.97 ppm, respectively), indicating that this FLP‐P‐Bi‐Co_3_O_4_ electrocatalyst interacts with both C1 and C2 carbons of GLY through the Bi center. The results obtained from this ^13^C NMR investigation are consistent with those acquired by FTIR.

### 2.7 A Proposed Reaction Mechanism for the FLP‐P‐Bi‐Co_3_O_4_‐Catalytic GOR

Based on the above spectroscopic analyses and catalytic tests, the presence of the FLP‐P‐Bi‐Co_3_O_4_ interface is the key factor responsible for the specific catalytic oxidation of GLY to DHA under the base‐free conditions. In Figure 8c, we propose a reaction mechanism for the GLY oxidation on the FLP‐P‐Bi‐Co_3_O_4_ surface. The reaction is initiated by the oxidation of a water molecule on the Co center of FLP‐P‐Bi‐Co_3_O_4_ to produce the Co‐OOH intermediate (detected by in situ Raman spectroscopy in Figure 6a,b), which is then stabilized by the neighboring phosphate groups modified on the FLP surface (Figure 5). Meanwhile, GLY coordinates with the Bi center of FLP‐P‐Bi‐Co_3_O_4_, where the secondary hydroxyl group of GLY interacts with the Bi center stronger than the primary counterpart, leading to the predominant activation of the secondary carbon of GLY (Figure 8a,b). The subsequent abstraction of the secondary (or primary) hydrogen atom(s) from GLY via the reaction with Co‐OOH results in the concurrent formation of DHA (or GLD). Finally, the oxidation of GLD gives rise to the formation of GLA and FA (Figure 7d).

## 3 Conclusion

In this study, we introduce a hybrid electrocatalyst composed of FLP‐P nanosheets and Bi‐Co_3_O_4_ platelets to simultaneously promote the effective cathodic HER and anodic OER intermediate‐assisted selective oxidation of GLY to DHA in a neutral aqueous environment. The FLP‐P‐Bi‐Co_3_O_4_ electrocatalyst is designed to significantly enhance the selectivity of GLY oxidation to DHA through addressing several key factors. The Co center in the FLP‐P‐Bi‐Co_3_O_4_ electrocatalyst facilitates the generation of the OER intermediates, such as Co‐OH and Co‐OOH. The phosphate groups modified on FLP stabilize the Co‐OOH intermediate to increase the overall electrocatalytic efficiency. This dual functionality of the FLP‐P‐Bi‐Co_3_O_4_ electrocatalyst − facilitating both formation and stabilization of the Co‐OOH intermediate − is called a bifunctional mechanism. The Bi center of the FLP‐P‐Bi‐Co_3_O_4_ electrocatalyst preferentially coordinates with the secondary hydroxyl group of GLY. This selective interaction helps to control the reaction pathway and direct the oxidation process toward DHA, minimizing the formation of undesired products. The electron transfer from FLP‐P to Bi‐Co_3_O_4_ creates additional active sites (Co^2+^) that are beneficial for OER, indirectly supporting the selective oxidation of GLY to DHA. This increase in active sites allows for more efficient catalysis, further promoting the product selectivity toward high‐value chemicals. With the assistance of operando Raman spectroscopy in the FLP‐P‐Bi‐Co_3_O_4_‐catalytic water splitting, we were able to identify the key intermediates (i.e., Co‐OH and Co‐OOH) during OER and confirm the reaction mechanism based on experimental observations. Taking advantage of in situ Raman spectroscopic detection, we optimized the FLP‐P‐Bi‐Co_3_O_4_‐catalytic GOR by tuning the electrochemical potential for the maximum production of the Co‐OOH intermediate. Consequently, an extraordinary electrocatalytic activity was achieved by successfully converting GLY (85% in conversion) to DHA (89% in selectivity).

## 4 Experimental Section

### Synthesis of FLP Nanosheets from Bulk BP

FLP nanosheets were prepared from the liquid‐phase exfoliation of bulk BP using NMP as a solvent. In a typical process, bulk BP crystal was grounded into small sizes with a mortar pestle. The grounded BP crystal (10 mg) was added to degassed NMP (20 mL) and then stirred in an argon (Ar) atmosphere for 6 h to intercalate the NMP solvent molecules into the BP interlayers for better exfoliation. Subsequently, the FLP‐containing solution was ultrasonicated for 2 h using a probe sonicator. During the ultrasonication for dispersion, the temperature was kept below 10 °C controlled by an ice bath. Finally, the dispersion was centrifuged at 1000 rpm for 5 min to remove the non‐exfoliated bulk BP.

### Surface Modification of Phosphate Groups on FLP Nanosheets

The as‐prepared FLP nanosheets were oxidized in air at 50 °C for 30 min to form FLP‐P nanosheets.

### Synthesis of Bi‐Co_3_O_4_ Platelets

In the synthesis of Bi‐Co_3_O_4_ platelets, 0.01 m Bi(NO_3_)_3_·5H_2_O and 0.1 m Co(NO_3_)_2_·6H_2_O were added to 5 mL of a 0.5 m HNO_3_ solution. Subsequently, 2 mL of 0.2 m NaOH in 5 mL deionized water was added to the above mixture. This mixture was stirred at room temperature for 2 h and then dried at 80 °C. Finally, the obtained powder of Bi‐Co_3_O_4_ platelets was calcined at 350 °C for 4 h.

### Synthesis of the FLP‐Bi‐Co_3_O_4_ Hybrid

In a typical synthesis process of FLP‐Bi‐Co_3_O_4_, 10 mg of BP was grounded to small pieces, followed by adding 20 mL of NMP and stirring in an Ar atmosphere for 2 h. The solution was then ultrasonicated for 2 h using a probe sonicator. Subsequently, the FLP‐containing solution was centrifuged at 1000 rpm for 5 min to remove the non‐exfoliated bulk BP. Afterward, 10 mg of the as‐synthesized Bi‐Co_3_O_4_ was added to the FLP‐containing solution and ultrasonicated for 30 min, followed by stirring the solution mixture in an Ar atmosphere for 12 h. After the reaction, the dispersion was filtered, washed with ethanol, and then heated at 80 °C in a nitrogen (N_2_) atmosphere for 1 h to remove the solvent.

### Synthesis of the FLP‐P‐Bi‐Co_3_O_4_ Hybrid

After obtaining the as‐prepared FLP‐Bi‐Co_3_O_4_, the FLP‐Bi‐Co_3_O_4_ sample was heated in air at 50 °C for 30 min to form the FLP‐P‐Bi‐Co_3_O_4_ hybrid.

### Spectroscopic and Electron Microscopic Characterizations

High‐resolution transmission electron microscopy (HR‐TEM) images of the as‐prepared samples were obtained in a TEM (JEOL, JEM‐2100F) with an acceleration voltage of 200 kV. Raman spectra of the as‐synthesized electrocatalysts were recorded in a micro‐Raman spectrometer (Horiba, LabRAM HR Evolution), equipped with a grating of 1800 grooves mm^−1^, a detector (Jobin Won Horiba, SDrive‐500 Syncerity), and a 532 nm laser system including an optical microscope (Olympus, CX41) and a cryostat (Linkam‐LTS 420). Fourier‐transform infrared (FTIR) spectra were collected in a spectrophotometer (Horiba, FT720). X‐ray photoelectron spectroscopy (XPS) measurements were conducted in an ESCA photoelectron spectrometer (Ulvac‐PHI 1600) with the photon energy of 1486.6 ± 0.2 eV (Al Kα radiation). The binding energies of the observed XPS signals were calibrated against the carbon C 1s peak at 284.8 eV. The Shirley‐Sherwood method was employed to subtract the background.

Chelating experiments for different electrocatalysts with GLY were carried out by mixing GLY with different electrocatalysts. After stirring for 5 h at room temperature, the mixture was filtered, and the sample was analyzed by FTIR and ^13^C NMR.

### Electrochemical Measurements

Electrochemical tests for OER, HER, and GOR were conducted using an Autolab electrochemical workstation in an H‐type three‐electrode cell at room temperature. The two compartments of the H‐type cell were separated by a proton exchange membrane (Nafion). The electrodes used in electrochemical measurements include an Ag/AgCl electrode as a reference electrode, a platinum (Pt) wire as a counter electrode, and the as‐prepared electrode as a working electrode. The working electrode was fabricated on a carbon cloth substrate. Before the electrocatalytic experiments, the carbon cloth substrates were cleaned sequentially with acetone, 1 m HNO_3_, and deionized water to thoroughly remove surface impurities. The electrode material was deposited onto a 1 × 1 cm^2^ area of the carbon cloth using a drop‐casting method and then dried under vacuum at 60 °C. A 0.5 m Na_2_SO_4_ aqueous solution at pH 7.0 was used as the electrolyte for experiments. All electrochemical potentials mentioned in this work have been converted to the reversible hydrogen electrode (RHE, in volts) scale according to:

(1) $$E_{\mathrm{RHE}}=E_{\mathrm{Ag}/\mathrm{AgCl}}+0.195+0.059\;\mathrm{pH}$$

The performance of the working electrodes was evaluated using current versus potential (I–V) scans, chronoamperometry, cyclic voltammetry (CV), and electrochemical impedance spectroscopy (EIS). EIS measurements were conducted under open‐circuit conditions over a frequency range of 10^−2^ to 10^5^ Hz. GOR was carried out in a 0.5 m Na_2_SO_4_ solution containing 0.1 m GLY. The exposed area of the working electrode in the electrolyte was maintained at 1 × 1 cm^2^, and all current densities were normalized to the geometrical surface area of the electrode.

The selectivity of a specific product is calculated by:

(2) $$selectivity\;\left(\%\right)=\frac{moles\;of\;the\;specific\;product}{moles\;of\;all\;products}\;\times 100$$

The Faradaic efficiency (*FE*) of products is calculated based on the following equation:

(3) $$FE=\frac{n\times c\times V\times F}{Q}\times 100\%$$

where *n* is the number of the required charges to oxidize GLY to a specific product molecule; *c* is the product concentration (mol L^−1^); *V* is the volume of the electrolyte solution; *F* is the Faraday constant (96485 C mol^−1^); *Q* is the total charge passed during the electrolysis reaction.

## Conflict of Interest

The authors declare no conflict of interest.

## Author Contributions

S.K.P. performed conceptualization, investigation, formal analysis, data curation, and wrote the original draft. H.J. performed investigation and formal analysis. C.C.C. performed investigation. K.W.S. performed visualization and supervision. Y.‐F.C. performed visualization and supervision. Y.‐T.C. performed supervision, conceptualization, funding acquisition, project administration, and reviewed and edited the final manuscript.

## Supporting information



Supporting Information

## Data Availability

The data that support the findings of this study are available from the corresponding author upon reasonable request.
