# Supplementary material for: Glycerol Electro‐Oxidation to Dihydroxyacetone with Coupled Hydrogen Production via In Situ Optimization of Water Oxidation Intermediates
Source: Small. 2025 Aug 4;21(38):e04892. doi: 10.1002/smll.202504892 (PMC12462562; doi:10.1002/smll.202504892)
Supplement: Supplementary file 1 — Supporting Information [file SMLL-21-e04892-s001.docx]

Supporting Information

**Glycerol electrooxidation to dihydroxyacetone with coupled hydrogen production via in situ optimization of water oxidation intermediates**

*Sandip K. Pahari, Heng Jao, Chih-Chian Chang, Kuan-Wei Su*, Yung-Fu Chen*, Yit-Tsong Chen**

**Table of Contents**

|  | **Title** | **Page** |
| --- | --- | --- |
|  | Supplementary methods | S4-6 |
| **Figure S1.** | A diagram of the possible reaction pathways in the GLY oxidation reaction | S7 |
| **Figure S2.** | The TEM and HR-TEM images of FLP and Co_3_O_4_ | S7 |
| **Figure S3.** | The TEM, HR-TEM, and SEM images together with EDS elemental mappings of FLP-Bi-Co_3_O_4_ and FLP-P-Co_3_O_4_ | S8 |
| **Figure S4.** | The P 2p core level XPS spectrum of FLP nanosheets | S9 |
| **Figure S5.** | The UV-Vis diffuse reflectance spectra and Tauc’s plots of Bi-Co_3_O_4_ and FLP-P | S9 |
| **Figure S6.** | The Mott-Schottky plots of FLP-P and Bi-Co_3_O_4_ | S10 |
| **Figure S7.** | The Co 2p core level XPS spectra of FLP-P-Bi-Co_3_O_4_ and FLP-Bi-Co_3_O_4_ | S10 |
| **Figure S8.** | The nitrogen adsorption isotherm plots of FLP-P-Bi-Co_3_O_4_ and FLP-Bi-Co_3_O_4_ | S11 |
| **Figure S9.** | The specific current density vs. scan rate plots with the current density taken from the non-Faradic region of the CV plot at 0.1 V | S11 |
| **Figure S10.** | The LSV curves normalized respectively to the geometric, BET, and ECSA surface areas | S13 |
| **Figure S11.** | Comparison of the EIS curves between the FLP-P-Bi-Co_3_O_4_ and FLP-Bi-Co_3_O_4_ electrocatalysts | S13 |
| **Figure S12.** | Calibrated curves of the GLY oxidation products obtained from the HPLC chromatograms of standard chemicals with known concentrations | S14 |
| **Figure S13.** | HPLC analysis for the products from an FLP-P-Bi-Co_3_O_4_-assisted GLY oxidation reaction | S14 |
| **Figure S14.** | A comparison of the GLY conversion and product selectivity distribution | S15 |
| **Figure S15.** | Comparison of the Raman spectra of an FLP-P-Bi-Co_3_O_4_ electrode before and after used in GOR for five reaction cycles | S16 |
| **Figure S16.** | The polarization curve of the FLP-P-Bi-Co_3_O_4_-assisted GOR in a two-electrode system | S16 |
| **Figure S17**  **–S19.** | The FTIR investigations for the interactions of GLY with different electrocatalysts | S17 |
| **Figure S20**  **–S22.** | The ^13^C NMR investigations for the interactions of GLY with different electrocatalysts | S18–19 |
| **Table 1.** | Comparison of the HER performance between FLP-P-Bi-Co_3_O_4_ and other reported electrocatalysts in neutral medium | S20 |
| **Table 2.** | Comparison of the OER performance between FLP-P-Bi-Co_3_O_4_ and other reported electrocatalysts in neutral medium | S21 |
| **Table 3.** | Product selectivity in the GLY oxidation reactions catalyzed by various electrocatalysts | S22 |
| **Table 4.** | Comparison of the GOR performance between FLP-P-Bi-Co_3_O_4_ and other reported electrocatalysts | S23-24 |
|  | References | S24-25 |

**Supplementary methods**

**Experimental Section**

**Materials**

GLY (C_3_H_8_O_3_, 99%), dihydroxyacetone (C_3_H_6_O_3_, 99%), DL-glyceric Acid (C_3_H_6_O_4_, 95%), and formic acid (HCO_2_H, 98%) were obtained from Tokyo Chemical Industry Co., Ltd. Cobalt nitrate (Co(NO_3_)_2_·6H_2_O), bismuth nitrate (Bi(NO_3_)_3_·5H_2_O), sulfuric acid (H_2_SO_4_, 98%), sodium sulfate (Na_2_SO_4_, 99%), acetonitrile (CH_3_CN, 99.9%), ammonium hydroxide, N-methyl-2-pyrrolidone (NMP), and black phosphorous (BP) were purchased from Sigma. All reagents used were of the analytical reagent grade. Deionized water (Millipore, Milli-Q grade) was used in all experiments.

**Synthesis of Co_3_O_4_ platelets**

In the synthesis of Co_3_O_4_ platelets, 0.1 M Co(NO_3_)_2_·6H_2_O was added to 5 mL of a 0.5 M HNO_3_ solution. Subsequently, 2 mL of 0.2 M NaOH in 5 mL deionized water was added to the above mixture. This mixture was stirred at room temperature for 2 hours and then dried at 80 °C. Finally, the obtained powder of Co_3_O_4_ platelets was calcined at 350 °C for 4 hours.

**Synthesis of the FLP-Co_3_O_4_ hybrid**

In a typical synthesis process of FLP- Co_3_O_4_, 10 mg of BP was grounded to small pieces, followed by adding 20 mL of NMP and stirring in an Ar atmosphere for 2 hours. The solution was then ultrasonicated for 2 hours using a probe sonicator. Subsequently, the FLP-containing solution was centrifuged at 1,000 rpm for 5 minutes to remove the non-exfoliated bulk BP. Afterwards, 10 mg of the as-synthesized Co_3_O_4_ was added to the FLP-containing solution and ultrasonicated for 30 minutes, followed by stirring the solution mixture in an Ar atmosphere for 12 hours. After the reaction, the dispersion was filtered, washed with ethanol, and then heated at 80 ^o^C in a nitrogen (N_2_) atmosphere for 1 hour to remove the solvent.

**Synthesis of the FLP-P-Co_3_O_4_ hybrid**

After obtaining the as-prepared FLP-Co_3_O_4_, the FLP-Co_3_O_4_ sample was heated in air at 50 °C for 30 minutes to form the FLP-P-Co_3_O_4_ hybrid.

**Product quantification**

During the chronopotentiometry test for the GLY oxidation process, while the sample solution was collected, the liquid products were separated and analyzed by high performance liquid chromatography (HPLC, Ultimate 3000, Thermo Fisher Scientific) equipped with a photodiode array detector. The detection wavelength was set at 210 nm. For the sample separation, a ChromCore Sugar-10H column was used as the stationary phase. The mobile phase was 5 mM H_2_SO_4_ (98%, HPLC grade) prepared with Milli-Q water. The flow rate was 0.5 mL/min with a column temperature of 60 °C and the injection volume was 20 μL. For each product, a calibration curve was obtained using a known concentration of various compounds.

Faradaic efficiency (*FE*) of the electrochemical GLY oxidation was calculated based on the following balanced half-reactions, corresponding to the conversion of GLY into individual oxidized products. If the stoichiometric coefficient for a given product is >1 in the balanced reaction (e.g., 1 GLY will produce 3 formic acids (FA)), the number of electrons for that half-reaction is divided by the stoichiometric coefficient to give the number of electrons required to form 1 mole of that product. This number of electrons is then used in the *FE* calculation

Glycerol → DHA

C_3_H_8_O_3_ + 2 OH^–^ → C_3_H_6_O_3_ + 2H_2_O + 2 e^–^ e^–^ count for DHA = 2e^–^

Glycerol → GLD

C_3_H_8_O_3_ + 2 OH^–^ → C_3_H_6_O_3_ + 2H_2_O + 2 e^–^ e^–^ count for DHA = 2e^–^

Glycerol → GLA

C_3_H_8_O_3_ + 4OH^–^ → C_3_H_6_O_4_ + 4H_2_O + 4e^–^ e^–^ count for GLA = 4e^–^

Glycerol → FA

C_3_H_8_O_3_ + 8OH^–^ → 3CO_2_H_2_ + 5H_2_O + 8e^–^ e^–^ count for FA = 8/3e^–^

**Chelating experiments for different electrocatalysts with GLY**

**Procedures for FTIR**

Fourier-transform infrared spectroscopy (FTIR) measurements were conducted in the ATR-FTIR (Attenuated Total Reflectance-FTIR) setup using a Bruker Vertex80v spectrometer equipped with a Ge ATR crystal and a DTGS KBr detector. A small aliquot of GLY (approximately 1-2 drops) was applied directly onto the surface of the ATR crystal. Spectral data were collected over the range of 4000–600 cm⁻¹ with a resolution of 2 cm⁻¹, and 200 scans were averaged per sample to improve the signal-to-noise ratio. A background spectrum was recorded prior to each sample measurement to account for atmospheric contributions. For the sample preparation, 1 mL GLY was desolve in 10 mL water solution. For the chelation experiment with different electrocatalysts, a 10 mL water solution of GLY and a 5 mg electrocatalyst were stirred for 5 hours at room temperature. Subsequently, the solution was filtered and analyzed with ATR-FTIR.

**Procedures for NMR**

For the NMR analysis, the process was similar to a reported article with some modification. [S1] A nuclear magnetic resonance spectrometer (JEOL, ECZ500R/S1) equipped with a superconducting magnet of the magnetic field of 11.74 Tesla (500 MHz) was used for NMR analysis. For NMR spectroscopy, tetramethylsilane (TMS) is the standard reference compound used to define the chemical shift scale. For the chelation experiment with different electrocatalysts, a 10 mL deuterated water solution of GLY (1 mL GLY was disolve in 10 mL D_2_O) and a 5 mg electrocatalyst were stirred for 5 hours at room temperature. Subsequently, the solution was filtered and analyzed with NMR.

*
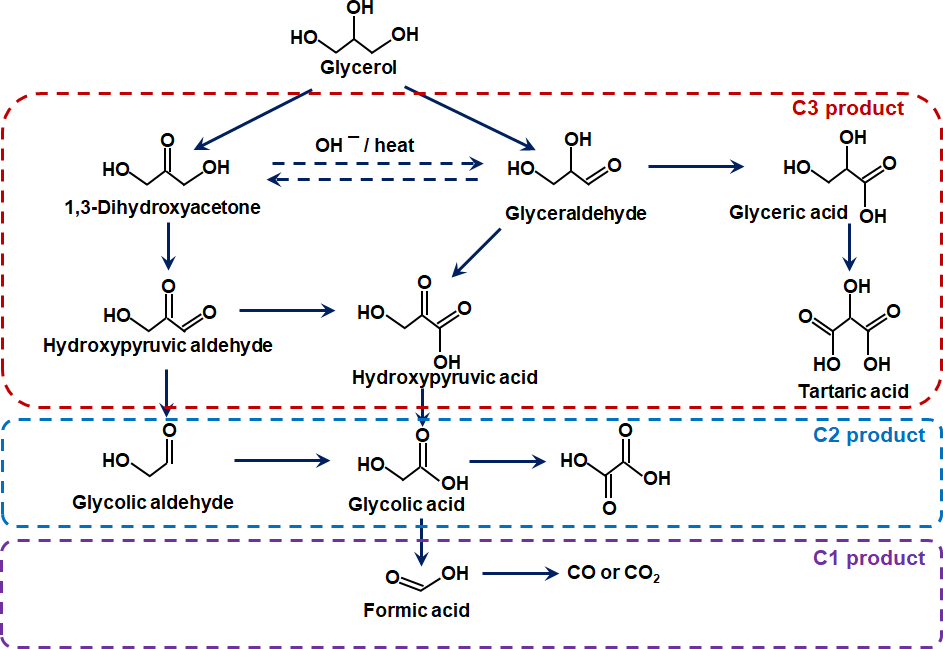
*

**Figure S1.** A diagram of the possible reaction pathways in the GLY oxidation reaction


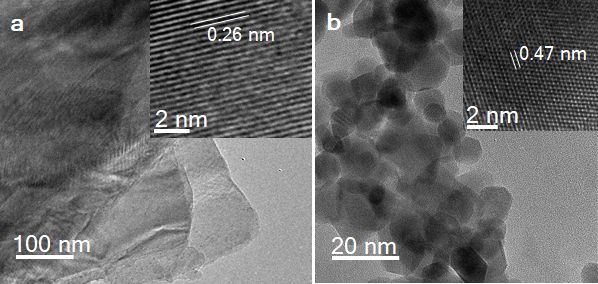


**Figure S2.** The TEM and HR-TEM images of FLP and Co_3_O_4_

a) The TEM and HR-TEM (in the inset) images of FLP nanosheets.

b) The TEM and HR-TEM (in the inset) images of Co_3_O_4_ platelets.


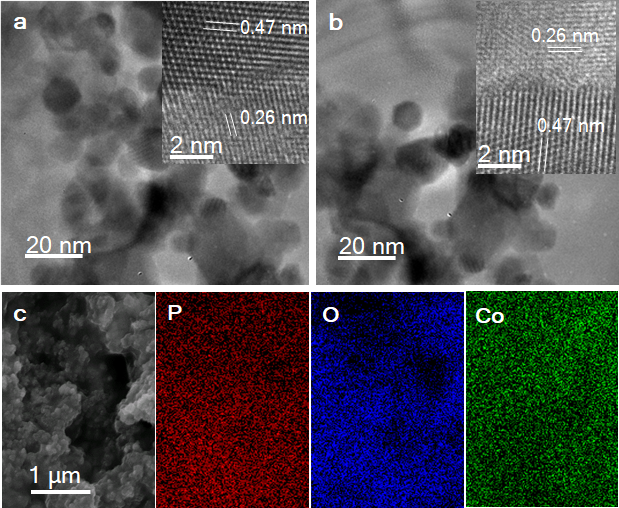


**Figure S3.** The TEM, HR-TEM, and SEM images together with EDS elemental mappings of FLP-Bi-Co_3_O_4_ and FLP-P-Co_3_O_4_

a) The TEM and HR-TEM (in the inset) images of FLP-Bi-Co_3_O_4_.

b) The TEM and HR-TEM (in the inset) images of FLP-P-Co_3_O_4_.

c) The SEM image of FLP-P-Co_3_O_4_ together with its EDS elemental mappings of phosphorous (P, red), oxygen (O, blue), and cobalt (Co, green).

**
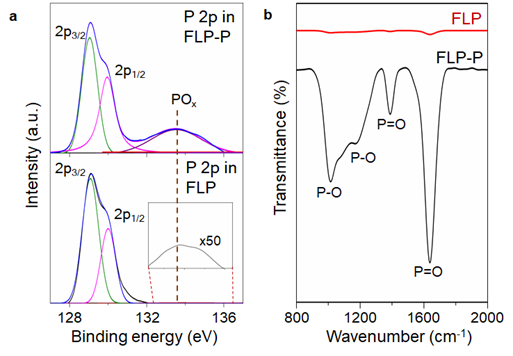
**

**Figure S4.** The P 2p core level XPS and FTIR spectra of FLP and FLP-P nanosheets

a) The P 2p core level XPS spectrum of FLP and FLP-P nanosheets.

b) The FTIR spectra of FLP and FLP-P nanosheets confirm the presence of phosphate groups functionalized on FLP-P.


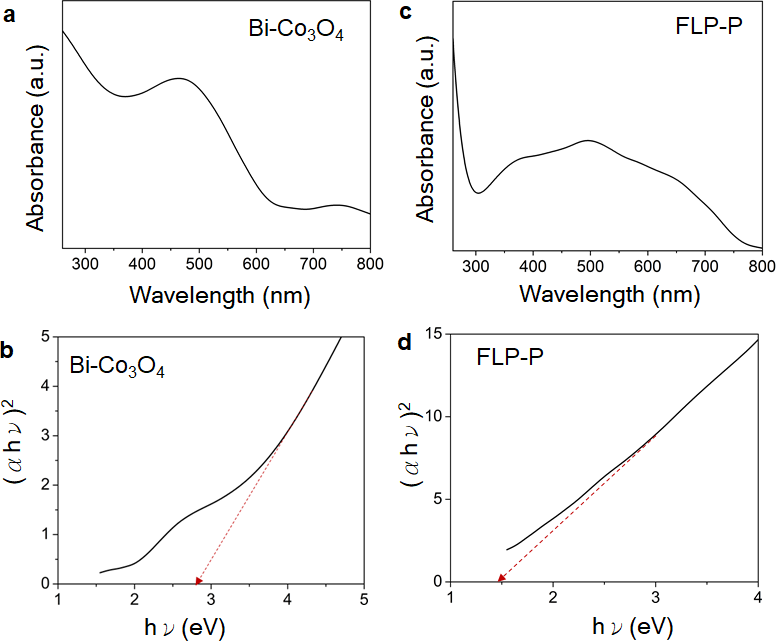


**Figure S5.** The UV-Vis diffuse reflectance spectra and Tauc’s plots of Bi-Co_3_O_4_ and FLP-P

a) The UV-Vis diffuse reflectance spectrum of Bi-Co_3_O_4_.

b) The Tauc’s plot of Bi-Co_3_O_4_.

c) The UV-Vis diffuse reflectance spectrum of FLP-P.

d) The Tauc’s plot of FLP-P.


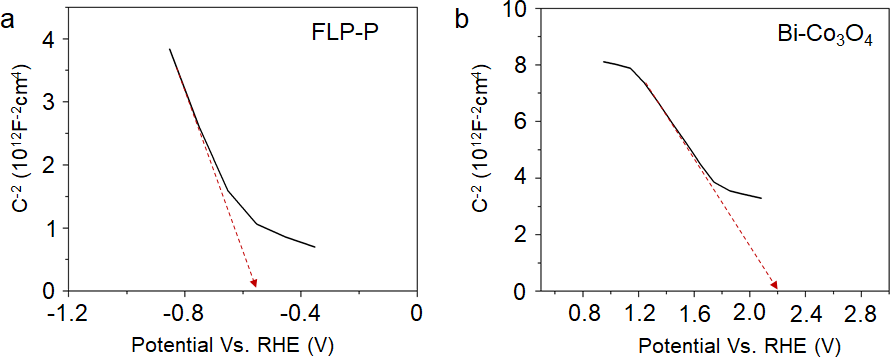


**Figure S6.** The Mott-Schottky plots of FLP-P and Bi-Co_3_O_4_

a) The Mott-Schottky plot of FLP-P.

b) The Mott-Schottky plot of Bi-Co_3_O_4_.


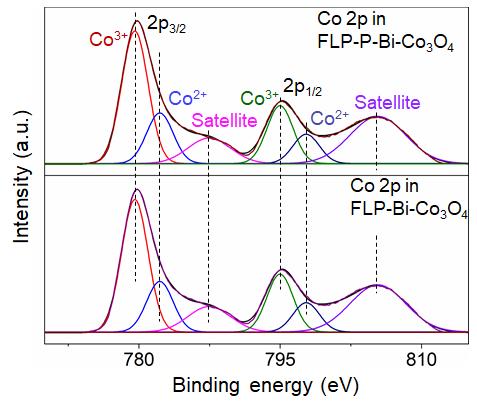


**Figure S7.** The Co 2p core level XPS spectra of FLP-P-Bi-Co_3_O_4_ and FLP-Bi-Co_3_O_4_


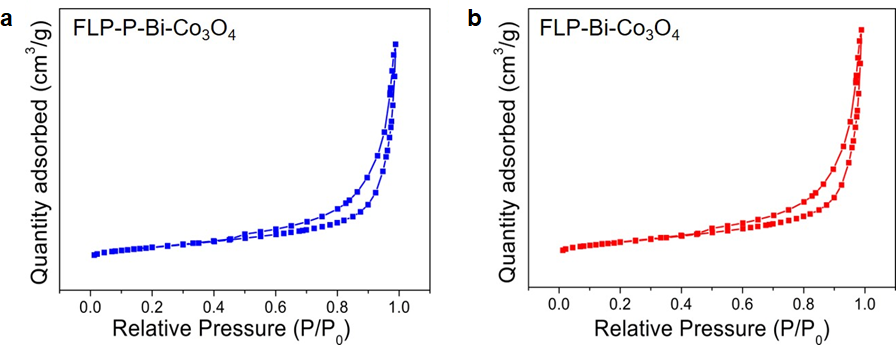


**Figure S8.** The nitrogen adsorption isotherm plots of FLP-P-Bi-Co_3_O_4_ and FLP-Bi-Co_3_O_4_

a) The nitrogen adsorption isotherm plot of FLP-P-Bi-Co_3_O_4_.

b) The nitrogen adsorption isotherm plot of FLP-Bi-Co_3_O_4_.


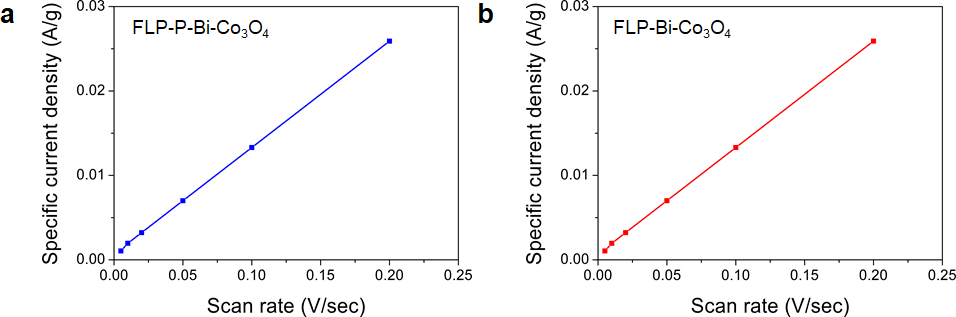


**Figure S9.** The specific current density vs. scan rate plots with the current density taken from the non-Faradic region of the CV plot at 0.1 V

a) Using the FLP-P-Bi-Co_3_O_4_ electrocatalyst.

b) Using the FLP-Bi-Co_3_O_4_ electrocatalyst.

The electrochemical surface area (ECSA) of a catalyst was determined by evaluating the electrochemical double-layer capacitance (*C_dl_*) of the sample. This capacitance was quantified by measuring the non-Faradaic capacitive current associated with the double-layer charging, as inferred from the scan-rate dependence of cyclic voltammograms (CV). The ECSA was subsequently calculated using the relation

*ECSA = C_dl_/C_s_*

where *C_dl_* denotes the electrochemical double-layer capacitance of the sample, and *C_s_* represents the specific capacitance of a standard electrode material with a unit surface area. In this study, the literature-reported value of 0.02 mF/cm² for the *C_s_* of carbon-based electrode materials was used in the ECSA calculation. The double-layer charging current (*i_c_*) is related to *C_dl_* and the scan rate (*ν*)

*i_c_ = ν·C_dl_*

Consequently, plotting the *i_c_* vs. *ν* curve (Figure **S9**) yields a linear relationship, with the slope corresponding to *C_dl_*. Accordingly, the ECSAs of the FLP-P-Bi-Co_3_O_4_ and FLP-Bi-Co_3_O_4_ electrodes were determined to be 5.25 and 5.29 m²/g, respectively.


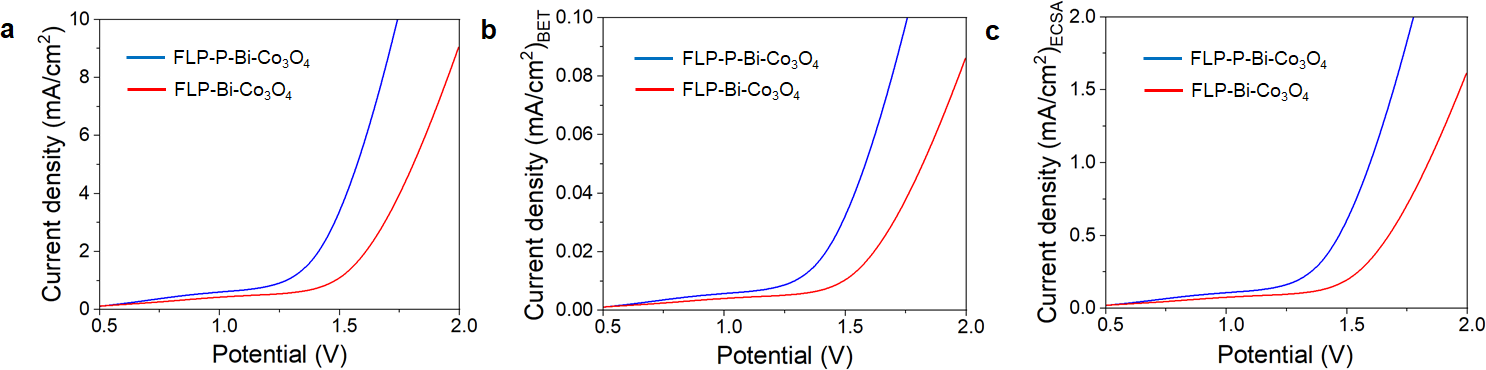


**Figure S10.** The LSV curves normalized respectively to the geometric, BET, and ECSA surface areas

a) The LSV curves normalized to the geometric surface areas.

b) The LSV curves normalized to the BET surface areas.

c) The LSV curves normalized to the ECSA surface areas.


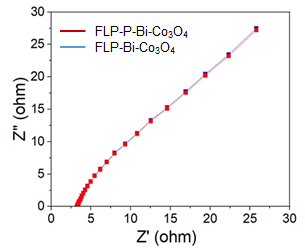


**Figure S11.** Comparison of the EIS curves between the FLP-P-Bi-Co_3_O_4_ and FLP-Bi-Co_3_O_4_ electrocatalysts

**
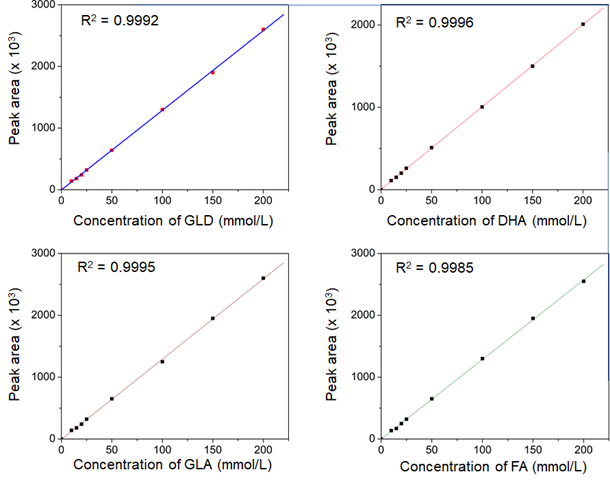
**

**Figure S12.** Calibrated curves of the GLY oxidation products obtained from the HPLC chromatograms of standard chemicals with known concentrations

*
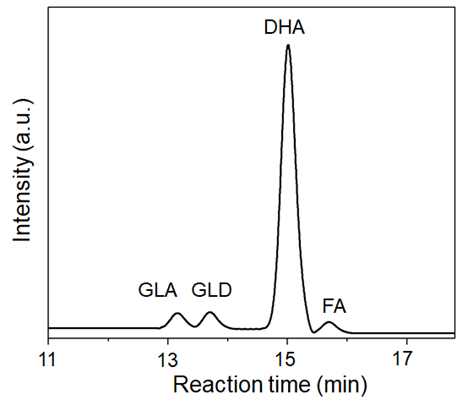
*

**Figure S13.** HPLC analysis for the products from an FLP-P-Bi-Co_3_O_4_-assisted GLY oxidation reaction

**
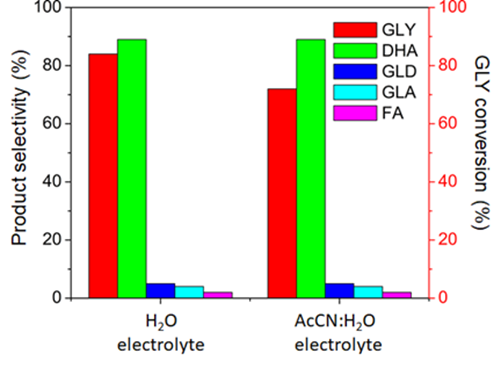
**

**Figure S14.** A comparison of the GLY conversion and product selectivity distribution resulting from the use of 0.5 M Na_2_SO_4_ containing 0.1 M GLY (represented as an H_2_O electrolyte) or 0.5 M Na_2_SO_4_ dissolved in the AcCN:H_2_O (5:1) solvent (represented as an AcCN:H_2_O electrolyte)


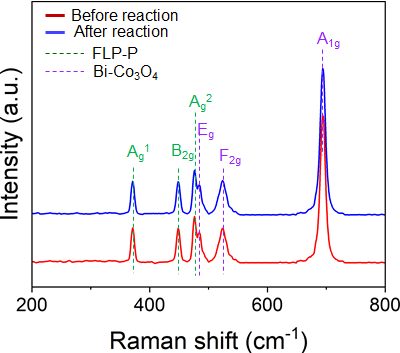


**Figure S15**. Comparison of the Raman spectra of an FLP-P-Bi-Co_3_O_4_ electrode before and after used in GOR for five reaction cycles


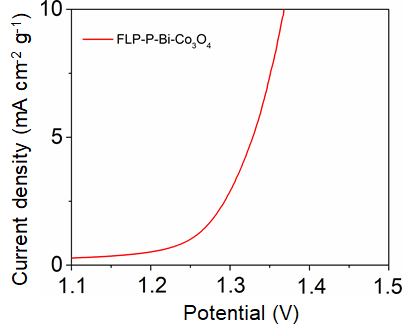


**Figure S16.** The polarization curve of the FLP-P-Bi-Co_3_O_4_-assisted GOR in a two-electrode system


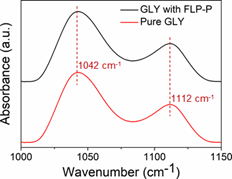


**Figure S17.** The FTIR investigation of pure GLY as against GLY in the presence of the FLP-P electrocatalyst


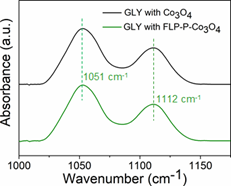


**Figure S18.** The FTIR investigation of GLY in the presence of the FLP-P-Co_3_O_4_ or Co_3_O_4_-electrocatalyst indicating that FLP-P does not influence the coordination of GLY with Co


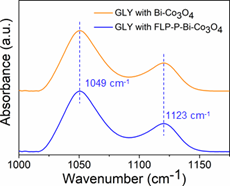


**Figure S19.** The FTIR investigation of GLY in the presence of the FLP-P-Bi-Co_3_O_4_ or Bi-Co_3_O_4_-electrocatalyst indicating that FLP-P does not influence the coordination of GLY with Bi


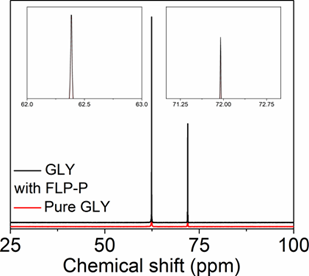


**Figure S20.** The ^13^C NMR investigation of pure GLY as against GLY in the presence of the FLP-P electrocatalyst


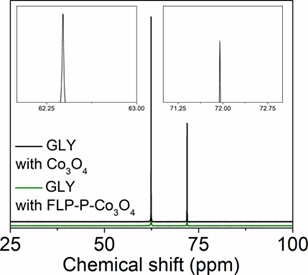


**Figure S21.** The ^13^C NMR spectrum of the coordination of GLY with the Co center in the FLP-P-Co₃O₄ or Co₃O₄ electrocatalyst revealing that FLP-P does not affect the coordination of GLY with Co


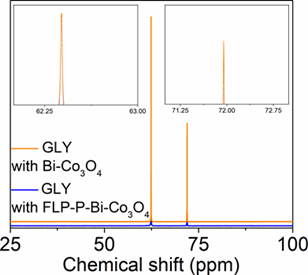


**Figure S22.** The ^13^C NMR spectrum of the coordination of GLY with the Bi center in the FLP-P-Bi-Co₃O₄ or Bi-Co₃O₄ electrocatalyst revealing that FLP-P does not affect the coordination of GLY with Bi

**Table S1.** Comparison of the HER performance between FLP-P-Bi-Co_3_O_4_ and other reported electrocatalysts in neutral medium

| Electrocatalyst | Electrolyte | Overpotential (mV) at 10 mA/cm^2^ | Ref. |
| --- | --- | --- | --- |
| FLP-P-Bi-Co_3_O_4_ | 0.5 M Na_2_SO_4_ | 88 | This work |
| CoSe_2_/CoP | 1 M PBS | 185 | Adv. Mater. 2022, 34,  2110631. |
| NiCo_2_P_x_ | 1 M PBS | 173 | Adv. Mater. 2017,  29, 1605502. |
| Fe_3_W_3_C NRs/RGO | 0.5 M K_2_SO_4_ | 103 | Nano Energy. 2019, 62, 85-93. |
| Fe@N–CNT/IF | 0.5 M Na_2_SO_4_ | 525 | Adv. Sci. 2019, 6, 1901458. |

**Table S2.** Comparison of the OER performance between FLP-P-Bi-Co_3_O_4_ and other reported electrocatalysts in neutral medium

| Electrocatalyst | Electrolyte | Overpotential (mV) at 10 mA/cm^2^ | Ref. |
| --- | --- | --- | --- |
| FLP-P-Bi-Co_3_O_4_ | 0.5 M Na_2_SO_4_ | 166 | This work |
| NiCo@NC | 1.0 M PBS | 396 | Int. J. Hydrog. Energy. 2020, 46, 8871-8884. |
| Fe@N–CNT/IF | 0.5 M Na_2_SO_4_ | 525 | Adv. Sci. 2019, 6, 1901458. |
| Ru-VO_2_ | 1 M PBS | 269 | Adv. Mater. 2024, 36, 2310690 |
| Bi_2_WO_6_ CNPs | 0.5 M Na_2_SO_4_ | 370 | J. Mater. Chem. A 2016, 4, 2438-2444. |
| (Ni,Co)_3_O_4_-RuO_2_ hybrid | 0.55 M sodium  phosphate | 270 | ACS Appl. Energy Mater. 2024, 7, 4445−4453. |
| IrCoOx/C | 0.5 M KHCO_3_ | 470 | Appl. Catal. B 2020, 269, 118820. |
| CoP@CoOOH | 1 M PBS | 318 | Small 2022, 18, 2106012. |
| CoIr-0.2 | 1 M PBS | 373 | Adv. Mater. 2018, 30, 1707522. |
| Co(OH)2 | 0.4 M KBi + 1.5 M  KF + 0.5 M KHCO_3_ | 421 | Chem 2022, 8, 2700–2714. |
| Cu-CoP | 1.0 M PBS | 411 | Appl. Catal. B. 2019, 11, 118555. |
| Ni-CoOOH | 1.0 M PBS | 410 | Chem. Eng. J., 2020, 8, 125537. |

**Table S3.** Product selectivity in the GLY oxidation reactions catalyzed by various electrocatalysts

| Electrocatalysts | GLY conversion (%) | DHA selectivity (%) | GLD selectivity (%) | GLA selectivity (%) | FA selectivity (%) |
| --- | --- | --- | --- | --- | --- |
| Co_3_O_4_ | 52 | 46 | 34 | 6 | 14 |
| Bi-Co_3_O_4_ | 61 | 74 | 12 | 8 | 6 |
| FLP-P-Bi-Co_3_O_4_ | 85 | 89 | 5 | 4 | 2 |

In Table S4, a comparison of the GOR performance between the FLP-P-Bi-Co_3_O_4_ of this work and other recently reported electrocatalysts is made with particular focus on energy efficiency and economic viability. Among these electrocatalysts, the FLP-P-Bi-Co₃O₄ demonstrates the highest reported selectivity for the DHA production at high current density. Notably, the reaction proceeds under neutral conditions without the need for a buffer solution and utilizes low-cost transition metal-based components to enhance the economic appeal of the process. Furthermore, the GOR achieves high current densities at low overpotentials, contributing to its overall energy efficiency.

**Table S4.** Comparison of the GOR performance between FLP-P-Bi-Co_3_O_4_ and other recently reported electrocatalysts

| **Catalyst** | **DHA Selectivity** | **Operating Conditions** | **Energy Efficiency** | **Economic Viability** | **Ref.** |
| --- | --- | --- | --- | --- | --- |
| FLP-P-Bi-Co₃O₄ | 89% | 10 mA cm^−2^ at 1.3 V_RHE_  0.5 M Na_2_SO_4_ | High current density; minimal OER competition | Low-cost (Co/Bi), scalable synthesis | This report |
| Co_3_O_4_ | 60 | 1.7 V_RHE_ 1.0 mA/cm^2^  0.1 M GLY & 0.1 M Na_2_B_4_O_7_ | Moderate overpotential; borate stabilization | Low-cost, non-precious metal, Borate additives increase operational costs | S2 |
| amorphous cobalt oxide | 46 | 1.7 V_RHE_  2.7 mA/cm^2^  0.1 M GLY & 0.1 M Na_2_B_4_O_7_ | Moderate overpotential; borate stabilization | Low-cost, non-precious metal, moderate selectivity, Borate additives increase operational costs | S3 |
| NiOOH | 71 | 1.52 V_RHE_ borate buffer in KOH (pH 9) | Moderate overpotential; borate stabilization | Low-cost, non-precious metal, moderate selectivity, Borate additives increase operational costs | S4 |
| PtBi/C | 58 | 0.82 V vs. RHE  4 mA/cm^2^  0.1 M GLY & 0.5 M H_2_SO_4_ | low overpotential; use of acid cause corrosion, side reactions, safety risks, environmental issues, | precious metal Prohibitively expensive | S5 |
| SbOx-Pt/OMC | 81.1 | 0.5 M H_2_SO_4_ solution | low overpotential; use of acid cause corrosion, side reactions, safety risks, environmental issues | precious metal Prohibitively expensive | S6 |
| MnO_2_-CuO/CF | 60 | 10 mA cm^−2^ at 1.3 V  1 M KOH | Moderate overpotential but Bases can cause electrode corrosion, handling hazards, and limited catalyst stability in alkaline media. | Low-cost, non-precious metal, moderate selectivity | S7 |
| CuO | 60 | 1.76 V_RHE_ 3 mA/cm^2^  0.1 M GLY &  0.1 M Na_2_B_4_O_7_ (pH 9) | high overpotential; borate stabilization | Low-cost, non-precious metal, moderate selectivity | S8 |
| Electro-Fenton Process at c-NiSe_2_ Cathode | 20.6 | 0.60 V_RHE_  50 mM GLY  0.1 M NaHSO_4_/  Na_2_SO4  (pH 2.85) | low overpotential; use of acid cause corrosion, side reactions, safety risks, environmental issues | Low-cost, non-precious metal, moderate selectivity | S9 |

**References**

[S1] S. Feng, J. Yi, H. Miura, N. Nakatani, M. Hada, T. Shishido, *ACS Catal*. **2020**, *10*, 11, 6071.

[S2] T.-G. Vo, P.-Y. Tsai, C.-Y. Chiang, *J. Catal.* **2023**, *424*, 64.

[S3] T.-G. Vo, P.-Y. Ho, C.-Y. Chiang, *Appl. Catal. B- Environ.* **2022**, *300*, 120723.

[S4] M. K. Goetz, M. T. Bender, K.-S. Choi, *Nat. Commun.* **2022**, *13,* 5848.

[S5] S Lee, H. J. Kim, E. J. Lim, Y. Kim, Y. Noh, G. W. Huber, W. B. Kim, *Green Chem.* **2016**, *18*, 2877-2887.

[S6] D. Kim, W.-G. Lim, Y. Kim, L. S. Oh, S. Kim, J. H. Park, Changshin Jo, Hyung Ju Kim, Joonhee Kang, Seonggyu Lee, Eunho Lim, *Appl. Catal. B- Environ.* **2023**, *339*, 123104.

[S7] Z. Huang, H. Ren, J. Guo, Y. Tang, D. Ye, J. Zhang, H. Zhao, *Appl. Catal. B- Environ.* **2024**, *351*, 123986.

[S8] C. Liu, M. Hirohara, T. Maekawa, R. Chang, T. Hayashi, C.-Y. Chiang, *Appl. Catal. B- Environ.* **2020**, *265*, 118543.

[S9] H. Sheng, A.N. Janes, R.D. Ross, H. Hofstetter, K. Lee, J.R. Schmidt, S. Jin, *Nat. Catal*. **2022**, *5*, 716.
